# Raman Spectroscopy on Free-Base Meso-tetra(4-pyridyl) Porphyrin under Conditions of Low Temperature and High Hydrostatic Pressure

**DOI:** 10.3390/molecules29102362

**Published:** 2024-05-17

**Authors:** Jhon Rewllyson Torres dos Reis, Fabio Furtado Leite, Keshav Sharma, Guilherme Almeida Silva Ribeiro, Welesson Henrique Natanael Silva, Alzir Azevedo Batista, Alexandre Rocha Paschoal, Waldeci Paraguassu, Mario Mazzoni, Newton Martins Barbosa Neto, Paulo Trindade Araujo

**Affiliations:** 1 Graduate Program in Physics, Institute of Natural Sciences, Federal University of Pará, Belém 66075-110, PA, Brazil; jhon.rewllyson@gmail.com (J.R.T.d.R.); fabioleite@unifap.br (F.F.L.); paraguassu@ufpa.br (W.P.); 2 Department of Exact and Technological Sciences, Federal University of Amapá, Macapá 68903-419, AP, Brazil; 3 Department of Physics and Astronomy, University of Alabama, Tuscaloosa, AL 35487, USA; ksharma1@crimson.ua.edu; 4 Department of Physics, Federal University of Minas Gerais, Belo Horizonte 31270-901, MG, Brazil; almeida.guilherme13@gmail.com (G.A.S.R.); welessonhenrique@gmail.com (W.H.N.S.); mmazzonibh@gmail.com (M.M.); 5 Departament of Chemistry, Federal University of São Carlos, São Carlos 13565-905, SP, Brazil; daab@ufscar.br; 6 Department of Physics, Federal University of Ceara, Fortaleza 60455-760, CE, Brazil; paschoal@fisica.ufc.br

**Keywords:** porphyrin, resonance Raman spectroscopy, hydrostatic pressure, low temperature

## Abstract

We present a Raman spectroscopy study of the vibrational properties of free-base meso-tetra(4-pyridyl) porphyrin polycrystals under various temperature and hydrostatic pressure conditions. The combination of experimental results and Density Functional Theory (DFT) calculations allows us to assign most of the observed Raman bands. The modifications in the Raman spectra when excited with 488 nm and 532 nm laser lights indicate that a resonance effect in the $Q_{y}$ band is taking place. The pressure-dependent results show that the resonance conditions change with increasing pressure, probably due to the shift of the electronic transitions. The temperature-dependent results show that the relative intensities of the Raman modes change at low temperatures, while no frequency shifts are observed. The experimental and theoretical analysis presented here suggest that these molecules are well represented by the $C_{2v}$ point symmetry group.

## 1. Introduction

Over the past few decades, porphyrin molecules have attracted a great deal of attention given their central role in numerous fundamental natural processes [1]. The interplay between the structural and spectroscopic properties of these molecules enables the optimization of their electronic characteristics aiming at specific applications [2,3].

**Figure 1:**
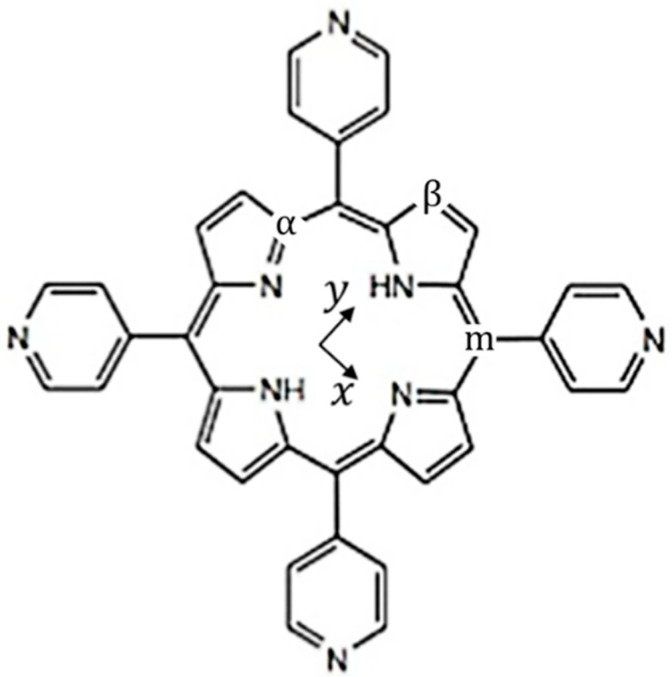
Schematic representation of free-base tetrapyridyl porphyrin (H_2_TPyP). Within the macrocycle’s plane, two distinct directions are defined: (i) the x-direction containing only nitrogen atoms, and (ii) the y-direction containing nitrogen atoms bonded with hydrogen. The indices α and β give the carbon positions in the pyrrolic rings, and m indicates the carbon position in the methynic bridge. The carbon atoms occupying these positions are labeled as follows: $C_{\alpha }$, linked to the central nitrogen atoms; $C_{\beta }$, located at the outer edge of the macrocycle; and $C_{m}$ (meso-carbon), connecting the pyrrolic rings.

The structure of porphyrins consists of a macrocycle formed by four pyrrolic rings interconnected with methyne bridges, and this arrangement is upheld by the insertion of either two hydrogen atoms (free-base porphyrins) or a metal ion (in metalloporphyrins) at the center of the macrocycle [1,4]; see Figure 1. The study of the optical properties associated with distinct porphyrins is driven by their cyclic conjugation, which leads to a pronounced absorption of near-ultraviolet and visible light as well as a red emission that is readily observable with the naked eye [1,4]. Additionally, these molecules present intriguing nonlinear optical traits [5,6]. Their absorption spectra are primarily composed of two characteristic bands known as the B-bands (or Soret bands), localized in the blue region of the spectrum, and the Q-bands, found in the green-red portion of the spectrum [1,4,7,8]. These spectroscopic responses are related to porphyrin’s electronic and vibronic properties, tuned through the modification of its structure, such as the substitution of the central ion and the addition of outlying and axial groups [3,6,7,8,9,10]. Those are very desirable possibilities since they create opportunities to employ porphyrin derivatives in many applications such as (i) photovoltaic cells [11,12,13], (ii) sensors [8,14], (iii) cancer treatment [15,16,17], and (iv) fluorescence imaging [18,19], among others.

Dissolved in organic solvents, free-base tetrapyridyl porphyrin (H_2_TPyP), as shown in Figure 1, depicts a complex Q-band with multiple electronic transitions and their corresponding vibronic progressions [20].

While the optical properties of tetrapyridyl porphyrins have been extensively studied [6,10,20,21], their vibrational properties, especially in their crystalline form, remain poorly explored. Although the vibrational modes of other porphyrins have been investigated [22,23,24,25], their behaviors are substantially different from H_2_TPyP’s vibrational modes. In addition, the few studies of H_2_TPyP modes lack proper assignments and detailed descriptions of their symmetries [26,27], which are intimately connected with porphyrin’s vibronic transitions [20]. In this context, Raman spectroscopy emerges as a non-invasive, fast, and reproducible method to study the properties of these vibrational modes under different thermodynamic conditions, e.g., low temperatures and high pressures [28,29,30,31,32,33]. 

In the present work, we combine Raman spectroscopy measurements with first-principle calculations to provide assignments for the Raman modes in poly-crystals of free-base tetrapyridyl porphyrin or C-H_2_TPyP (see Figure S1 in Supplementary Materials). The evolution of the assigned modes in C-H_2_TPyP under high pressures, low temperatures, and different excitation energies is addressed. In addition, we elucidate the modifications in porphyrin’s resonance conditions under high pressures, along with possible symmetry changes occurring at both high pressures and low temperatures.

## 2. Results and Discussion

### 2.1. Raman Bands Assignments 

**Figure 2:**
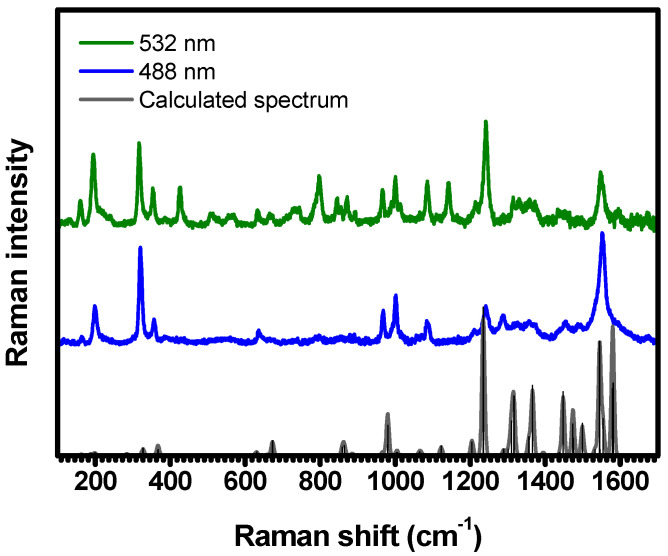
Raman spectra of C-H_2_TPyP experimentally obtained with excitation centered at 488 nm (represented by a blue solid line) and 532 nm (green solid line), and the DFT–calculated Raman spectrum for the H_2_TPyP molecule (gray solid line). In the theoretical spectrum, Raman intensity (in A^4^/amu) refers to the Raman activity (scattering factor).

The Raman spectra of C-H_2_TPyP show a rich distribution of bands, ranging from $150\;cm^{-1}$ to $1650\;cm^{-1}$; see Figure 2.

**Figure 3:**
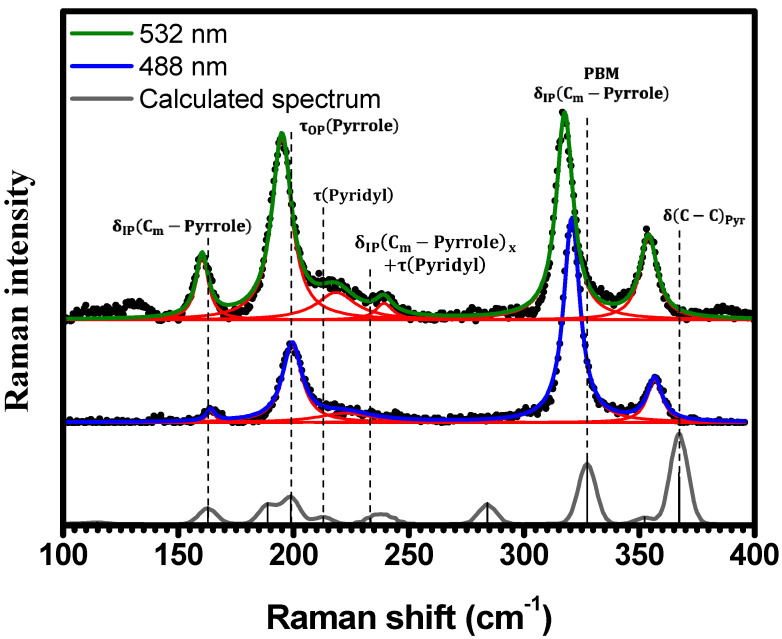
Experimental Raman spectra (top and middle) measured for C-H_2_TPyP (black dots) and calculated (bottom) for the H_2_TPyP molecule (gray solid line) under ambient conditions in the spectral range of $100\;{\;cm}^{-1}<\kappa <400\;{\;cm}^{-1}$. The experimental spectra were obtained by exciting the sample at 488 nm (middle spectrum) and 532 nm (top spectrum). The blue (488 nm) and green (532 nm) solid curves represent the fittings obtained through the deconvolution process using Lorentzian functions (red solid lines). In the theoretical spectrum, Raman intensity (in A^4^/amu) refers to the Raman activity (scattering factor).

**Figure 4:**
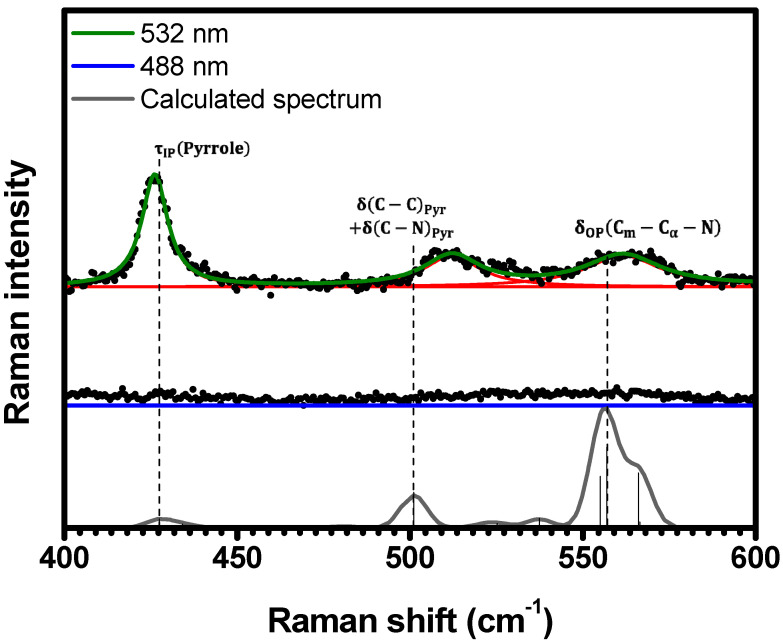
Experimental Raman spectra (top and middle) measured for C-H_2_TPyP (black dots) and calculated (bottom) for the H_2_TPyP molecule (gray solid line) under ambient conditions in the spectral range of $400\;cm^{-1}<\kappa <600\;cm^{-1}$. The experimental spectra were obtained by exciting the sample at 488 nm (middle spectrum) and 532 nm (top spectrum). The blue (488 nm) and green (532 nm) solid curves represent the fittings obtained through the deconvolution process using Lorentzian functions (red solid lines). In the theoretical spectrum, Raman intensity (in A^4^/amu) refers to the Raman activity (scattering factor).

**Figure 5:**
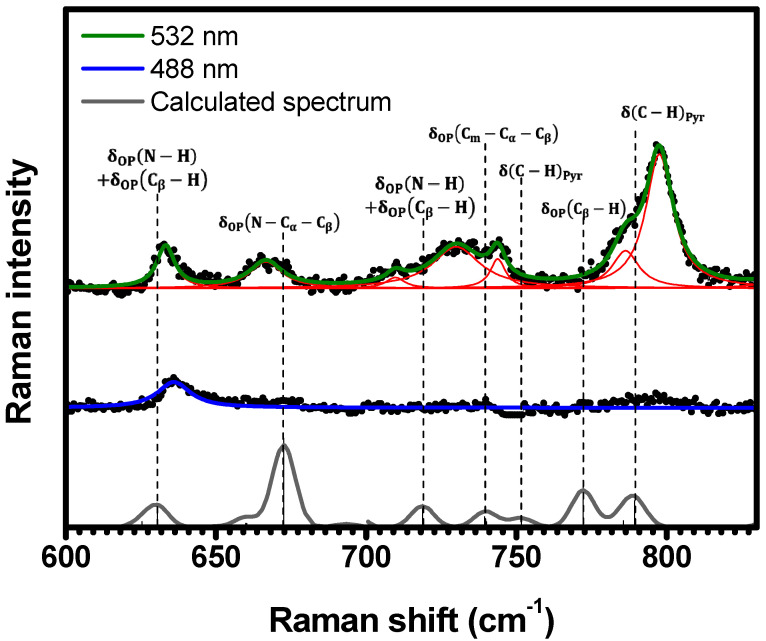
Experimental Raman spectra (top and middle) measured for C-H_2_TPyP (black dots) and calculated (bottom) for the H_2_TPyP molecule (gray solid line) under ambient conditions in the spectral range of $600\;cm^{-1}<\kappa <830\;cm^{-1}$. The experimental spectra were obtained by exciting the sample at 488  nm (middle spectrum) and 532  nm (top spectrum). The blue (488  nm) and green (532  nm) solid curves represent the fittings obtained through the deconvolution process using Lorentzian functions (red solid lines). In the theoretical spectrum, Raman intensity (in A^4^/amu) refers to the Raman activity (scattering factor).

**Figure 6:**
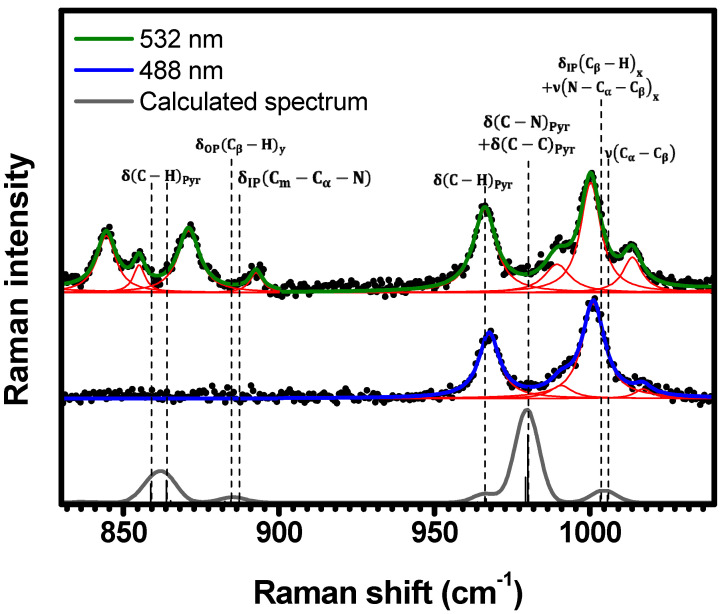
Experimental Raman spectra (top and middle) measured for C-H_2_TPyP (black dots) and calculated (bottom) for the H_2_TPyP molecule (gray solid line) under ambient conditions in the spectral range of $830\;cm^{-1}<\kappa <1040\;cm^{-1}$. The experimental spectra were obtained by exciting the sample at 488  nm (middle spectrum) and 532  nm (top spectrum). The blue (488  nm) and green (532 nm) solid curves represent the fittings obtained through the deconvolution process using Lorentzian functions (red solid lines). In the theoretical spectrum, Raman intensity (in A^4^/amu) refers to the Raman activity (scattering factor).

**Figure 7:**
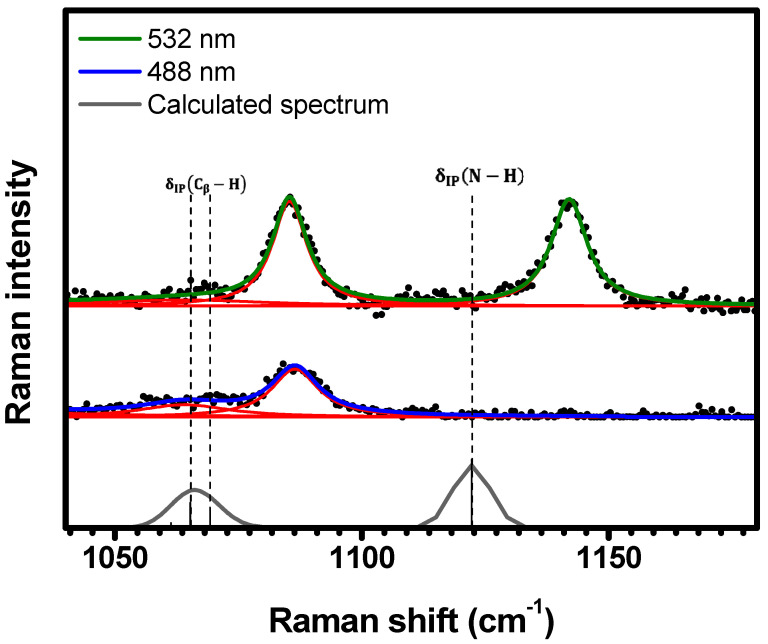
Experimental Raman spectra (top and middle) measured for C-H_2_TPyP (black dots) and calculated (bottom) for the H_2_TPyP molecule (gray solid line) under ambient conditions in the spectral range of $1040\;cm^{-1}<\kappa <1180\;cm^{-1}$. The experimental spectra were obtained by exciting the sample at 488 nm (middle spectrum) and 532 nm (top spectrum). The blue (488 nm) and green (532 nm) solid curves represent the fittings obtained through the deconvolution process using Lorentzian functions (red solid lines). In the theoretical spectrum, Raman intensity (in A^4^/amu) refers to the Raman activity (scattering factor).

**Figure 8:**
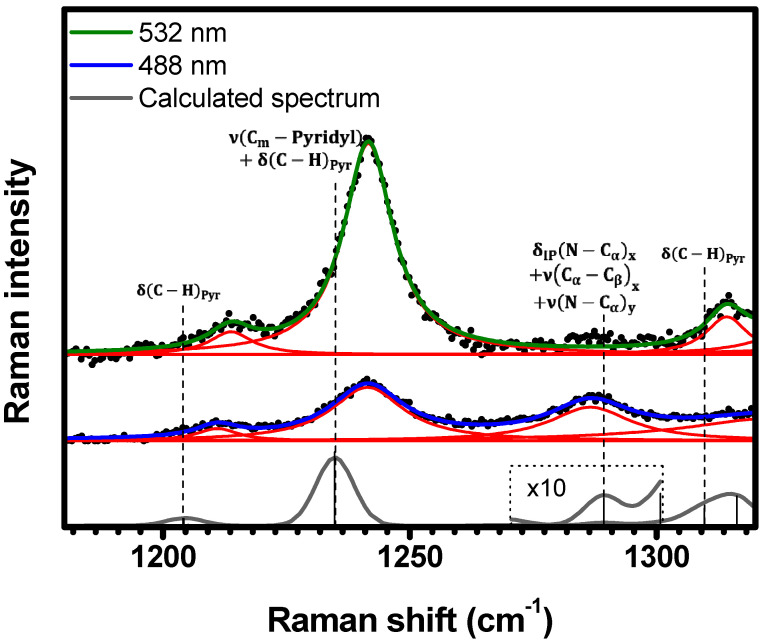
Experimental Raman spectra (top and middle) measured for C-H_2_TPyP (black dots) and calculated (bottom) for the H_2_TPyP molecule (gray solid line) under ambient conditions in the spectral range of $1180\;cm^{-1}<\kappa <1320\;cm^{-1}$. The experimental spectra were obtained by exciting the sample at 488 nm (middle spectrum) and 532 nm (top spectrum). The blue (488 nm) and green (532 nm) solid curves represent the fittings obtained through the deconvolution process using Lorentzian functions (red solid lines). In the theoretical spectrum, Raman intensity (in A^4^/amu) refers to the Raman activity (scattering factor).

**Figure 9:**
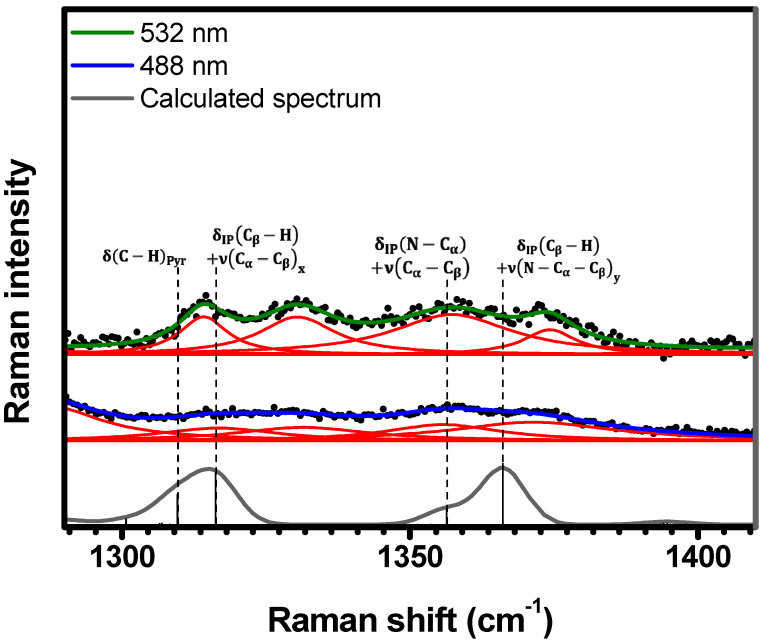
Experimental Raman spectra (top and middle) measured for C-H_2_TPyP (black dots) and calculated (bottom) for the H_2_TPyP molecule (gray solid line) under ambient conditions in the spectral range of $1290\;cm^{-1}<\kappa <1410\;cm^{-1}$. The experimental spectra were obtained by exciting the sample at 488 nm (middle spectrum) and 532 nm (top spectrum). The blue (488 nm) and green (532 nm) solid curves represent the fittings obtained through the deconvolution process using Lorentzian functions (red solid lines). In the theoretical spectrum, Raman intensity (in A^4^/amu) refers to the Raman activity (scattering factor).

**Figure 10:**
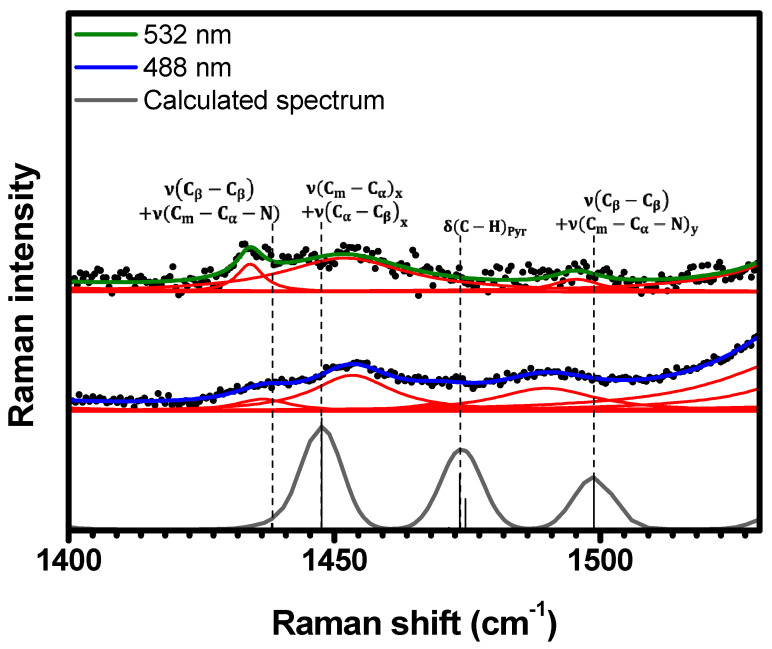
Experimental Raman spectra (top and middle) measured for C-H_2_TPyP (black dots) and calculated (bottom) for the H_2_TPyP molecule (gray solid line) under ambient conditions in the spectral range of $1400\;cm^{-1}<\kappa <1530\;cm^{-1}$. The experimental spectra were obtained by exciting the sample at 488 nm (middle spectrum) and 532 nm (top spectrum). The blue (488 nm) and green (532 nm) solid curves represent the fittings obtained through the deconvolution process using Lorentzian functions (red solid lines). In the theoretical spectrum, Raman intensity (in A^4^/amu) refers to the Raman activity (scattering factor).

**Figure 11:**
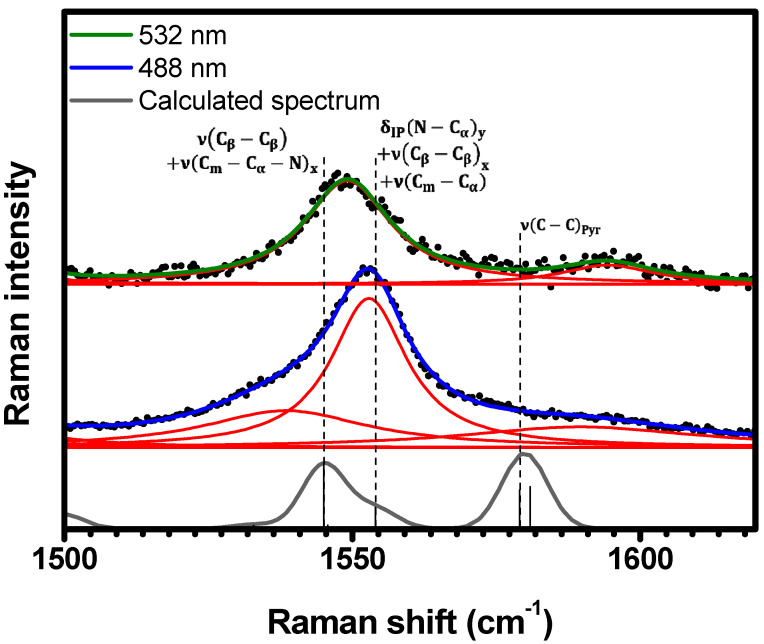
Experimental Raman spectra (top and middle) measured for C-H_2_TPyP (black dots) and calculated (bottom) for the H_2_TPyP molecule (gray solid line) under ambient conditions in the spectral range of $1500\;cm^{-1}<\kappa <1620\;cm^{-1}$. The experimental spectra were obtained by exciting the sample at 488 nm (middle spectrum) and 532 nm (top spectrum). The blue (488 nm) and green (532 nm) solid curves represent the fittings obtained through the deconvolution process using Lorentzian functions (red solid lines). In the theoretical spectrum, Raman intensity (in A^4^/amu) refers to the Raman activity (scattering factor).

These spectra were acquired by exciting the sample at 488 nm (resonant with the $Q_{y_{1}}(0,2)$ absorption band), and at 532 nm (resonant with the intersection between the $Q_{y1}(0,0)$ and $Q_{y2}(0,0)$ bands, referred to herein as the $Q_{y}(0,0)$ band) [20]. These vibronic progressions arise from the coupling of the electronic absorption band $Q_{y}(0,0)$ with the vibrational modes centered around $1245{\;cm}^{-1}$ ($Q_{y_{1}}(0,2)$ band) [20]. To provide a clearer depiction of the investigated modes, we present and discuss the results by zooming into the specific spectral regions, as depicted in Figure 3, Figure 4, Figure 5, Figure 6, Figure 7, Figure 8, Figure 9, Figure 10 and Figure 11. The experimental spectra were deconvoluted using Lorentzian functions. This constitutes a conventional approach in Raman spectroscopy, stemming from the intrinsic properties of Raman scattering (RS). The semi-classical interpretation of RS relies on the forced damped oscillator model, which follows the Lorentzian function. In the quantum mechanical framework, each vibration exhibits a Lorentzian probability of light scattering [34]. The fitting procedure enables us to identify the spectral band positions with a deviation of $\sim 0.2\;cm^{-1}$. The corresponding center-peak wavenumbers (κ) of the Raman bands, obtained with 488 nm and 532 nm, are listed in Table 1. Illustrations with an overview of the identified vibration patterns are provided in Table S2 in Supplementary Materials.

In Figure 3, the spectral region of $100{\;cm}^{-1}<\kappa <400{\;cm}^{-1}$ is displayed. Five Raman bands are observed for both excitations and are located at $164{\;cm}^{-1}$ ($161{\;cm}^{-1}$), $199{\;cm}^{-1}$ ($195{\;cm}^{-1}$), $223{\;cm}^{-1}$ ($221{\;cm}^{-1}$), $321{\;cm}^{-1}$ ($317{\;cm}^{-1}$), and $357{\;cm}^{-1}$ ($354{\;cm}^{-1}$) when excited at 488 nm (532 nm). A Raman band at $239{\;cm}^{-1}$ is observed in the spectrum obtained with 532 nm excitation, presenting no corresponding band in the spectrum obtained with 488  nm. Within this same spectral region, theoretical calculations predict nine Raman-active vibrational modes for the H_2_TPyP molecule. These modes are assigned to the following vibrations (OP stands for out-of-plane, and IP stands for in-plane): $\delta_{IP}\left(C_{m}-Pyrrole\right)$ at $163{\;cm}^{-1}$; $\nu \left(C_{m}-Pyridyl\right)$ at $189{\;cm}^{-1}$; $\tau_{OP}\left(Pyrrole\right)$ at $199{\;cm}^{-1}$; $\tau \left(Pyridyl\right)$ at $213{\;cm}^{-1}$; $\delta_{IP}{\left(C_{m}-Pyrrole\right)}_{x}+\tau \left(Pyridyl\right)$ at $233{\;cm}^{-1}$; $\tau \left(Pyrrole\right)$ at $284{\;cm}^{-1}$; $\delta_{IP}\left(C_{m}-Pyrrole\right)$ at $327{\;cm}^{-1}$; τ(Pyrrole) at $352{\;cm}^{-1}$; and $\delta {\left(C-C\right)}_{Pyr}$ at $367{\;cm}^{-1}$. Despite the shifts when compared to experimental results, the calculations indicate that the vibrations at $163\;cm^{-1}$, $199{\;cm}^{-1}$, $213{\;cm}^{-1}$, $233\;\;cm^{-1}$, $327{\;cm}^{-1}$, and $367{\;cm}^{-1}$ correspond to the six observed Raman bands, as shown in Figure 3 and summarized in Table 1 and Table S2 in SI (illustrations 1–6). No Raman bands were observed below $150{\;cm}^{-1}$ under ambient conditions or either excitation wavelengths. We note that the in-phase $\delta_{IP}\left(C_{m}-Pyrrole\right)$ mode at $327{\;cm}^{-1}$, from now on designated as “Pophyrin’s Breathing Mode (PBM)”, represents the breathing of porphyrin’s central ring.

As depicted in Figure 3, it is evident that the spectrum acquired with excitation at 532 nm exhibits greater resolution compared to the spectrum acquired with excitation at 488 nm. This observation aligns with the fact that the absorbance at 532 nm is approximately twice that at 488 nm [20], potentially resulting in a stronger resonance effect.

Within the spectral range of $400{\;cm}^{-1}<\kappa <600{\;cm}^{-1}$, no Raman bands were detected in the spectrum at 488 nm, as shown in Figure 4. Nevertheless, at 532 nm, three distinct Raman bands emerge at $426{\;cm}^{-1}$, $511{\;cm}^{-1}$, and $561{\;cm}^{-1}$, indicating the resonance of these modes with $Q_{y}(0,0)$ electronic transition. The observed Raman bands are assigned to the vibrations $\tau_{IP}\left(Pyrrole\right)$ at $427{\;cm}^{-1}$, $\delta {\left(C-C\right)}_{Pyr}+\delta {\left(C-N\right)}_{Pyr}$ at $501\;cm^{-1}$, and $\delta_{OP}\left(C_{m}-C_{\alpha }-N\right)$ at $557{\;cm}^{-1}$ in the calculated spectrum, respectively; see Table 1 and Table S2 in SI (illustrations 7–9).

Figure 5 presents the Raman spectra within the range of $600\;cm^{-1}<\kappa <830\;cm^{-1}$. Several Raman bands are resonant when porphyrins are excited under 532 nm ($633\;cm^{-1}$, $666{\;cm}^{-1}$, $710{\;cm}^{-1}$, $730{\;cm}^{-1}$, $744{\;cm}^{-1}$, $786{\;cm}^{-1}$, and $797{\;cm}^{-1}$), whereas the spectrum obtained with 488 nm excitation exhibits only one Raman band at $636\;cm^{-1}$ (corresponding to $633\;cm^{-1}$ at 532 nm). According to DFT calculations (see Table 1 and Table S2 in SI (illustrations 10–16)), these Raman bands are assigned to the following vibrations: $\delta_{OP}\left(N-H\right)+\delta_{OP}\left(C_{\beta }-H\right)$ at $630{\;cm}^{-1}$, $\delta_{OP}\left(N-C_{\alpha }-C_{\beta }\right)$ at $672{\;cm}^{-1}$, $\delta_{OP}\left(N-H\right)+\delta_{OP}\left(C_{\beta }-H\right)$ at $719{\;cm}^{-1}$, $\delta_{OP}\left(C_{m}-C_{\alpha }-C_{\beta }\right)$ at $739{\;cm}^{-1}$, $\delta {\left(C-H\right)}_{Pyr}$ at $752{\;cm}^{-1}$, $\delta_{OP}\left(C_{\beta }-H\right)$ at $772{\;cm}^{-1}$, and $\delta {\left(C-H\right)}_{Pyr}$ at $789{\;cm}^{-1}$. 

The spectral region of $830\;cm^{-1}<\kappa <1040\;cm^{-1}$ (Figure 6) displays eight resonant Raman bands ($844{\;cm}^{-1}$, $855{\;cm}^{-1}$, $871{\;cm}^{-1}$, $892{\;cm}^{-1}$, $966\;cm^{-1}$, $989\;cm^{-1}$, $1000\;cm^{-1}$, and $1014\;cm^{-1}$) under 532 nm excitation. However, when excited under 488 nm, only the higher energy bands at $967\;cm^{-1}$, $991\;cm^{-1}$, $1001\;cm^{-1}$, and $1017\;cm^{-1}$ are resonant. The DFT calculations (see Table 1 and Table S2 in SI (illustrations 17–24)) suggest the following assignments to these bands: two $\delta {\left(C-H\right)}_{Pyr}$ at $859{\;cm}^{-1}$ and $864{\;cm}^{-1}$, $\delta_{OP}(C_{\beta }-H{)}_{y}$ at $884{\;cm}^{-1}$, $\delta_{IP}\left(C_{m}-C_{\alpha }-N\right)$ at $887{\;cm}^{-1}$, $\delta {\left(C-H\right)}_{Pyr}$ at $966\;cm^{-1}$, $\delta {\left(C-N\right)}_{Pyr}+\delta {\left(C-C\right)}_{Pyr}$ at $980\;cm^{-1}$, $\delta_{IP}{\left(C_{\beta }-H\right)}_{x}+\nu {\left(N-C_{\alpha }-C_{\beta }\right)}_{x}$ at $1003\;cm^{-1}$, and $\nu \left(C_{\alpha }-C_{\beta }\right)$ at $1006\;cm^{-1}$. 

As shown in Figure 7, the spectral region of $1040{\;cm}^{-1}<\kappa <1180{\;cm}^{-1}$ exhibits three resonant Raman bands under 532 nm excitation: $1068\;cm^{-1}$, $1085\;cm^{-1}$, and $1142{\;cm}^{-1}$. The spectrum acquired with 488 nm displays two bands at $1068\;cm^{-1}$ and $1086{\;cm}^{-1}$ (the same bands observed at 532 nm). These bands are assigned to the $\delta_{IP}\left(C_{\beta }-H\right)$ vibrations at $1065{\;cm}^{-1}$ and $1069{\;cm}^{-1}$, and the $\delta_{IP}\left(N-H\right)$ vibration at $1122\;cm^{-1}$, respectively; see Table 1 and Table S2 in SI (illustrations 25–27).

Figure 8 shows the spectral range of $1180{\;cm}^{-1}<\kappa <1320{\;cm}^{-1}$. In this range, three Raman bands are resonant under both 532 nm ($1213{\;cm}^{-1}$, $1241{\;cm}^{-1}$, and $1314{\;cm}^{-1}$), and 488 nm ($1211{\;cm}^{-1}$, $1241{\;cm}^{-1}$, and $1287{\;cm}^{-1}$) excitations. The lower energy bands $1213{\;cm}^{-1}$ and $1241{\;cm}^{-1}$ at 532 nm ($1211{\;cm}^{-1}$ and $1241{\;cm}^{-1}$ at 488 nm) are assigned to the calculated vibrations $\delta {\left(C-H\right)}_{Pyr}$ at $1206{\;cm}^{-1}$, and $\nu \left(C_{m}-Pyridyl\right)+\delta {\left(C-H\right)}_{Pyr}$ at $1235{\;cm}^{-1}$, respectively. The theoretical mode $\delta_{IP}{\left(N-C_{\alpha }\right)}_{x}+\nu {\left(C_{\alpha }-C_{\beta }\right)}_{x}+\nu {\left(N-C_{\alpha }\right)}_{y}$ at $1289{\;cm}^{-1}$ is assigned to the $1287{\;cm}^{-1}$ band at 488 nm, while the $\delta {\left(C-H\right)}_{Pyr}$ mode at $1310{\;cm}^{-1}$ is assigned to the $1314{\;cm}^{-1}$ band at 532 nm; see Table 1 and Table S2 in SI (illustrations 28–31).

The spectral range of $1290{\;cm}^{-1}<\kappa <1410{\;cm}^{-1}$ exhibits the same four resonant Raman bands under both 532 nm and 488 nm excitations (see Figure 9) centered at $1314{\;cm}^{-1}$ (the same $1314{\;cm}^{-1}$ band discussed above in Figure 8), $1330{\;cm}^{-1}$, $1357{\;cm}^{-1}$, and $1373{\;cm}^{-1}$. The main difference between the two spectra lay on the intensity (i.e., resonant effects) of the bands: the bands at 488 nm appear less structured when compared with the band at 532 nm. According to the DFT calculations, these bands are assigned to the following vibrations: $\delta {\left(C-H\right)}_{Pyr}$ at $1310{\;cm}^{-1}$, $\delta_{IP}\left(C_{\beta }-H\right)+\nu {\left(C_{\alpha }-C_{\beta }\right)}_{x}$ at $1316{\;cm}^{-1}$, $\delta_{IP}\left(N-C_{\alpha }\right)+\nu \left(C_{\alpha }-C_{\beta }\right)$ at $1356{\;cm}^{-1}$, and $\delta_{IP}\left(C_{\beta }-H\right)+\nu {\left(N-C_{\alpha }-C_{\beta }\right)}_{y}$ at $1366{\;cm}^{-1}$; see Table 1 and Table S2 in SI (illustrations 31–34).

The spectral range $1400{\;cm}^{-1}<\kappa <1530{\;cm}^{-1}$, shown in Figure 10, exhibits three common Raman bands for each excitation. At 532 nm (488 nm), these bands are centered at $1434{\;cm}^{-1}$ ($1436{\;cm}^{-1}$), $1451{\;cm}^{-1}$ ($1454{\;cm}^{-1}$), and $1495{\;cm}^{-1}$ ($1489{\;cm}^{-1}$). The DFT calculations indicate that these bands correspond to the following vibrations: $\nu \left(C_{\beta }-C_{\beta }\right)+\nu \left(C_{m}-C_{\alpha }-N\right)$ at $1438{\;cm}^{-1}$, $\nu {\left(C_{m}-C_{\alpha }\right)}_{x}+\nu {\left(C_{\alpha }-C_{\beta }\right)}_{x}$ at $1448{\;cm}^{-1}$, and $\nu \left(C_{\beta }-C_{\beta }\right)+\nu {\left(C_{m}-C_{\alpha }-N\right)}_{y}$ at $1499{\;cm}^{-1}$; see Table 1 and Table S2 in SI (illustrations 35, 36 and 38). The vibration $\delta {\left(C-H\right)}_{Pyr}$ at $1474\;cm^{-1}$ is only resonant at 488 nm (see Table 1 and Table S2 in SI (illustrations 35–38).)

As shown in Figure 11, the spectral range $1500{\;cm}^{-1}<\kappa <1620\;cm^{-1}$ exhibits three resonant Raman bands centered at $1538{\;cm}^{-1}$, $1553{\;cm}^{-1}$, and $1589{\;cm}^{-1}$ in the 488 nm spectrum. The lower energy Raman band ($1538{\;cm}^{-1}$) is absent in the 532 nm spectrum, while the other two are also resonant, with their centers ($1549{\;cm}^{-1}$ and $1594{\;cm}^{-1}$) slightly redshifted. These bands are assigned to the vibrations $\nu \left(C_{\beta }-C_{\beta }\right)+\nu {\left(C_{m}-C_{\alpha }-N\right)}_{x}$ at $1545{\;cm}^{-1}$, $\delta_{IP}{\left(N-C_{\alpha }\right)}_{y}+\nu {\left(C_{\beta }-C_{\beta }\right)}_{x}+\nu \left(C_{m}-C_{\alpha }\right)$ at $1554{\;cm}^{-1}$, and $\nu {\left(C-C\right)}_{Pyr}$ at $1581{\;cm}^{-1}$; see Table 1 and Table S2 in SI (illustrations 39–41).

Table 1 provides assignments for a total of forty-one vibrational modes, comprising fifteen that resonate exclusively with $Q_{y}(0,0)$ electronic transition, three resonating only with $Q_{y}(0,2)$ vibronic progression, and twenty-three that resonate with both. It is noteworthy that reference [20] elucidated, via the deconvolution of the absorbance UV-Vis spectrum, that the $Q_{y}(0,2)$ vibronic progression arises from the coupling between $Q_{y}(0,0)$ electronic transition and a vibrational mode centered at $1245\;cm^{-1}$. This mode closely aligns in energy with $\delta \left(C_{m}-Pyridyl\right)+\delta {\left(C-H\right)}_{pyr}$ ($1241\;cm^{-1}$) which resonates with both 488 nm ($Q_{y}(0,2)$) and 532 nm ($Q_{y}(0,0)$); see Figure 8 and Table 1.

### 2.2. Hydrostatic Pressure Experiments

**Figure 12:**
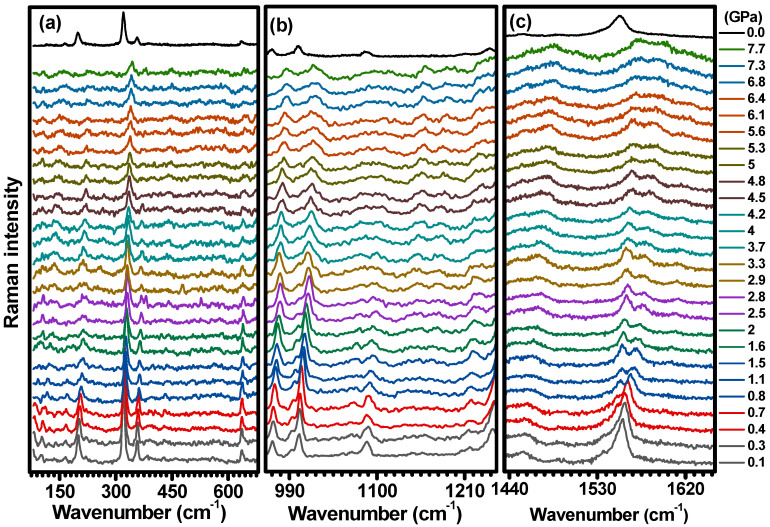
Raman spectrum of C-H_2_TpyP excited at 488 nm with pressures ranging from 0.1 Gpa to 7.7 Gpa for three distinct wavenumber regions: (**a**) $80–680{\;cm}^{-1}$, (**b**) $960–1250{\;cm}^{-1}$, and (**c**) $1435–1650{\;cm}^{-1}$. The spectrum at 0.0 GPA was acquired at ambient conditions and it is being shown as a reference.

To explore the structural stability of C-H_2_TPyP, studies were conducted under high-pressure conditions, from 0.1 GPa to 7.7 GPa. In Figure 12, the Raman spectra acquired from samples under ambient conditions and submitted to various loads are presented. To facilitate a more comprehensive discussion, the Raman spectra are divided into three distinct wavenumber regions: $80–680{\;cm}^{-1}$, $960–1250{\;cm}^{-1}$, and $1435–1650{\;cm}^{-1}$. It is worth noting that even at a very low pressure (0.1 GPa), some modes that were not visible under ambient conditions become apparent. The lack of theoretical prediction for some of these modes as vibrational modes of the H_2_TPyP molecule suggests that the bands in the low-wavenumber region ($100–150{\;cm}^{-1}$) are associated with the lattice vibration of C-H_2_TpyP, i.e., librations and the torsion of porphyrin’s ring.

**Figure 13:**
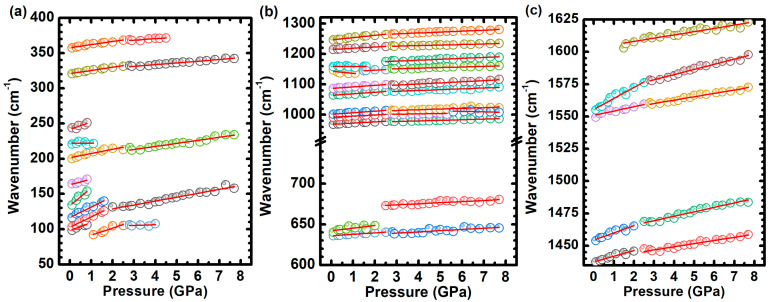
Evolutions of the frequencies of the Raman bands with increasing pressure. The evolution of the Raman bands with pressure is analyzed for three distinct wavenumber regions: (**a**) $50–400\;{\;cm}^{-1}$, (**b**) $600–1300{\;cm}^{-1}$, and (**c**) $1435–1625{\;cm}^{-1}$, to facilitate a more comprehensive discussion.

Figure 13 shows the evolution of the Raman band frequencies with increasing pressure, presenting distinct rates dω/dP, as summarized in Table 2. Notably, at pressures of 0.8  GPa, 1.5  GPa, 2.5  GPa, and 5.6  GPa, changes in the wavenumber displacement are evident for some Raman bands, as indicated in Table 2.

The crystal lattice frequency vibrations undergo high blueshift rates ($10.1–25\;\;cm^{-1}\;GPa^{-1}$). The vibrations initially at $98{\;cm}^{-1}$ and $133{\;cm}^{-1}$ disappear at 0.8 GPa, and the vibration initially at $117{\;cm}^{-1}$ disappears at 1.5 GPa. The remaining lattice vibrations at $103\;cm^{-1}$ and $81\;cm^{-1}$ have their shift rates decreased at 1.5 GPa and at 2.5  GPa, respectively. Moreover, the $\tau_{OP}(Pyrrole)$ vibration ($199{\;cm}^{-1}$) undergoes a blueshift at a rate of $3.8\;cm^{-1}\;GPa^{-1}$, manifesting a gradual decrease in intensity, followed by an increase in its Full Width at Half Maximum (FWHM). Both PBM ($323\;cm^{-1}$) and $\delta {\left(C-C\right)}_{Pyr}$ ($359\;cm^{-1}$) vibrations display a blueshift at comparable rates of $2.7\;cm^{-1}\;GPa^{-1}$ and $3.1\;cm^{-1}\;GPa^{-1}$, with $\delta {\left(C-C\right)}_{Pyr}$ observed until 4.5 GPa, while PBM remains up to 7.7 GPa. The $\delta_{IP}\left(C_{m}-Pyrrole\right)+\tau \left(Pyridyl\right)$ ($242{\;cm}^{-1}$) vibration undergoes a high blueshift rate ($10.1\;cm^{-1}\;GPa^{-1}$). In contrast, a minor displacement rate is identified for the $\delta_{OP}(N-H)+\delta_{OP}(C_{\beta }-H)$ ($636{\;cm}^{-1}$) mode ($1.3{\;cm}^{-1}{\;GPa}^{-1}$) throughout the entire process. This rate is notably lower when compared to the displacement rates of other vibrational modes within the range of 100 to $400{\;cm}^{-1}$. Furthermore, the disappearance of some lattice modes beyond 0.8 GPa and the observed increase in the FWHM bands suggest the initiation of an amorphization process.

In the region ranging from $960{\;cm}^{-1}$ and $1250{\;cm}^{-1}$, most of the Raman bands undergo a gradual blueshift, except for the band centered around $1144\;cm^{-1}$, which initially displays a redshift at a rate of $-8.8{\;cm}^{-1}{\;GPa}^{-1}$ (see Table 2 and Figure 13b). In the region within $1440{\;cm}^{-1}$ and $1650{\;cm}^{-1}$, the vibrations $\nu \left(C_{\beta }-C_{\beta }\right)+\nu \left(C_{m}-C_{\alpha }-N\right)$ ($1439{\;cm}^{-1}$), $\nu {\left(C_{m}-C_{\alpha }\right)}_{x}+\nu {\left(C_{\alpha }-C_{\beta }\right)}_{x}$ ($1456{\;cm}^{-1}$), $\delta_{IP}{\left(N-C_{\alpha }\right)}_{y}+\nu {\left(C_{\beta }-C_{\beta }\right)}_{x}+\nu \left(C_{m}-C_{\alpha }\right)\;$($1555{\;cm}^{-1}$), and $\nu {\left(C-C\right)}_{Pyr}$ ($1603{\;cm}^{-1}$) undergo a blueshift, presenting rates from $2.5{\;cm}^{-1}/\;GPa$ to $4.0\;{\;cm}^{-1}/\;GPa$. The vibrations $\nu \left(C_{\beta }-C_{\beta }\right)+\nu {\left(C_{m}-C_{\alpha }-N\right)}_{y}$ ($1489{\;cm}^{-1}$) and $\nu \left(C_{\beta }-C_{\beta }\right)+\nu {\left(C_{m}-C_{\alpha }-N\right)}_{x}$ ($1537{\;cm}^{-1}$) undergo an initial blueshift, with rates around $11\;{\;cm}^{-1}/\;GPa$. The former Raman band disappears at 0.8 GPa, and the latter has its shift rate greatly decreased at the same pressure. 

**Figure 14:**
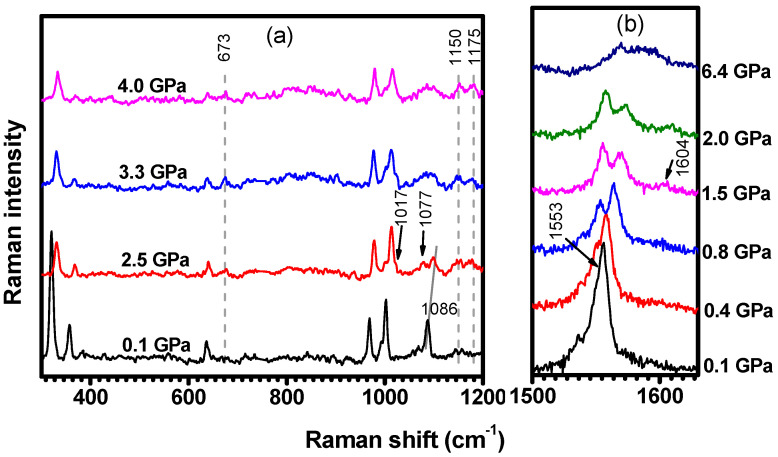
Raman spectra ranging from (**a**) $300\;{\;cm}^{-1}$ to $1200{\;cm}^{-1}$, and (**b**) from $1500{\;cm}^{-1}$ to $1800{\;cm}^{-1}$. The spectra were excited at 488 nm and acquired under different hydrostatic pressures.

Figure 14 shows representative C-H_2_TPyP Raman spectra for selected hydrostatic pressures. When compared with the spectrum at 0.1 GPa (the lowest pressure in the experiment), the spectrum at 2.5 GPa shows three new Raman modes centered at $673{\;cm}^{-1}$, $1150{\;cm}^{-1}$, and $1175{\;cm}^{-1}$; see Figure 14a. In addition, the intensity of the Raman mode at $1017{\;cm}^{-1}$ (see illustration 24 in Table S2 in Supplementary Materials) increases relative to the intensity of the mode at $1001\;cm^{-1}$ (see illustration 23 in Table S2 in Supplementary Materials), making both modes (see Table 1), which are associated with a distinct stretching of the carbons β and α, distinguishable. 

**Figure 15:**
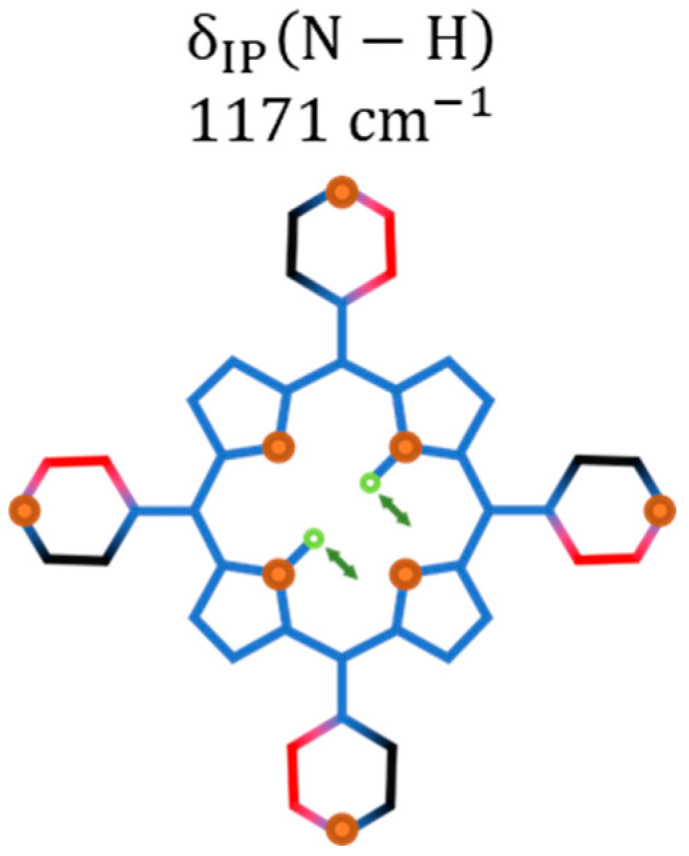
Schematic illustrations of the Raman vibration activated at higher pressures: bending of the N−H bonds; IP stands for in-plane.

The Raman band initially at $1086{\;cm}^{-1}$ (out-of-phase bending of the $C_{\beta }-H$ pair; see illustration 26 in Table S2 in Supplementary Materials) undergoes a frequency upshift and an intensity decrease, favoring the observation of the lower energy band at $1077\;cm^{-1}$ (in-phase bending of the $C_{\beta }-H$ pair; see illustration 25 in Table S2 in Supplementary Materials), whose main change is connected to its intensity increase. These two bands start fading and lose resolution when the pressure is further increased to 3.3 GPa. It is important to comment that the in-phase bending of the $C_{\beta }-H$ pair at $1077\;cm^{-1}$ appears at $1063\;cm^{-1}$ when measured at ambient conditions. With increasing pressure, the inactive vibration at ambient conditions $\delta_{IP}(N-H)$ centered at $1171\;cm^{-1}$ (see Figure 15) becomes active with the frequency slightly upshifted to $1175\;cm^{-1}$. It is worth mentioning that the modes centered at $673\;cm^{-1}$ and $1150\;cm^{-1}$ only undergo a slight enhancement of their intensities.

From Figure 12 and Figure 14b, it is noteworthy that the modes centered at $1537\;cm^{-1}$ ($\nu \left(C_{\beta }-C_{\beta }\right)+\nu {\left(C_{m}-C_{\alpha }-N\right)}_{x}$; see illustration 39 in Table S2 in Supplementary Materials) and at $1555{\;cm}^{-1}$ ($\delta_{IP}{\left(N-C_{\alpha }\right)}_{y}+\nu {\left(C_{\beta }-C_{\beta }\right)}_{x}+\nu \left(C_{m}-C_{\alpha }\right)$; see illustration 40 in Table S2 in Supplementary Materials) gradually upshift in frequency for pressures up to 7.7 GPa. Initially, $\nu \left(C_{\beta }-C_{\beta }\right)+\nu {\left(C_{m}-C_{\alpha }-N\right)}_{x}$ displays an higher upshift rate of $11.4\;cm^{-1}\;GPa^{-1}$ compared to the $7.5\;cm^{-1}\;GPa^{-1}$ observed for $\delta_{IP}{\left(N-C_{\alpha }\right)}_{y}+\nu {\left(C_{\beta }-C_{\beta }\right)}_{x}+\nu \left(C_{m}-C_{\alpha }\right)$. However, beyond 0.8 Gpa, both rates decrease to $2.9\;cm^{-1}\;GPa^{-1}$ and to $4.5\;cm^{-1}\;GPa^{-1}$, respectively. This implies that after 0.8 GPa, $\delta_{IP}{\left(N-C_{\alpha }\right)}_{y}+\nu {\left(C_{\beta }-C_{\beta }\right)}_{x}+\nu \left(C_{m}-C_{\alpha }\right)$ upshifts more than one and a half times when compared with $\nu \left(C_{\beta }-C_{\beta }\right)+\nu {\left(C_{m}-C_{\alpha }-N\right)}_{x}$. This observation explains the observed splitting in the Raman bands with increasing pressure. Their relative intensities present an interesting behavior: the intensity of the mode $\nu \left(C_{\beta }-C_{\beta }\right)+\nu {\left(C_{m}-C_{\alpha }-N\right)}_{x}$ is first enhanced and then suppressed with increasing pressure, while the intensity of the mode $\delta_{IP}{\left(N-C_{\alpha }\right)}_{y}+\nu {\left(C_{\beta }-C_{\beta }\right)}_{x}+\nu \left(C_{m}-C_{\alpha }\right)$ is continuously suppressed. Finally, the Raman-active vibration $\nu {\left(C-C\right)}_{Pyr}$ theoretically centered at $1581\;cm^{-1}$ (see illustration 41 in Table S2 in Supplementary Materials) undergoes both a frequency upshift to $1604\;cm^{-1}$ and a substantial enhancement of its intensity with increasing pressure. 

In addition to structural modifications observed, our findings indicate the influence of pressure load on resonance conditions of C-H_2_TPyP, probably due to modifications in the $Q_{y}(0,2)$ and $Q_{y}(0,0)$ bands’ energy gap. Indeed, as mentioned in Section 2.1, some of the Raman bands observed at 532 nm (488 nm) do not have a correspondent in the spectra at 488 nm (532 nm), evidencing the resonance effect [31,33,34], which occurs when the different regions of the optical absorption spectrum (i.e., the $Q_{y1}(0,2)$ and the $Q_{y}(0,0)$ bands) are excited [34]. It is also known that the resonance conditions of vibrational modes are often affected by external stimuli (e.g., temperature and pressure) that perturb the molecular geometry [35,36].

**Figure 16:**
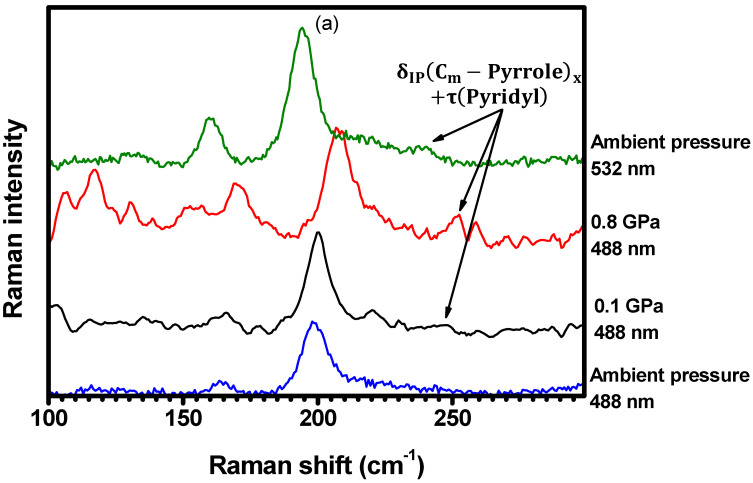

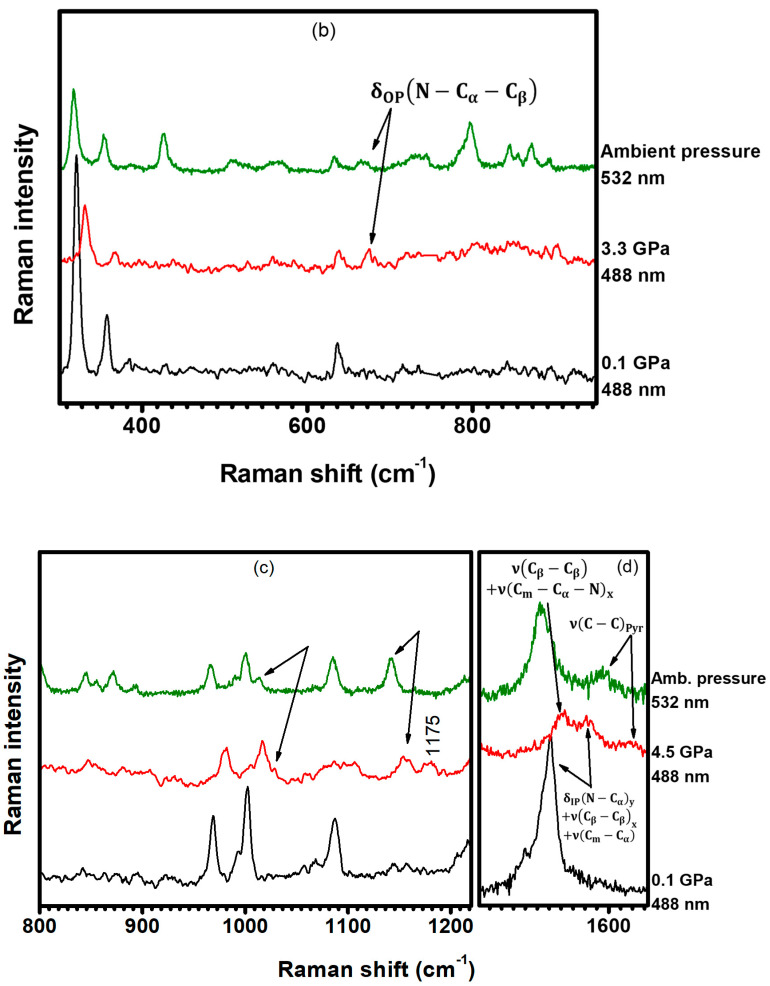
Raman spectra ranging from (**a**) $100\;cm^{-1}$ to $300\;cm^{-1}$, (**b**) $300{\;cm}^{-1}$ to $950{\;cm}^{-1}$, (**c**) $800{\;cm}^{-1}$ to $1200{\;cm}^{-1}$, and (**d**) from $1400{\;cm}^{-1}$ to $1700{\;cm}^{-1}$. The spectra were acquired under different hydrostatic pressures and excited at both 488 nm and 532 nm (ambient conditions).

A new Raman mode at $242\;cm^{-1}$ is observed at 0.1 GPa with excitation at 488 nm, as shown in Figure 16a, and its intensity increases with compression, up to 0.8 GPa. Furthermore, as shown in Figure 16b, the Raman-active vibration centered at $673{\;cm}^{-1}$ (not present in the spectrum at 0.1 GPa) has emerged in the spectrum acquired at 2.5 GPa with the excitation at 488 nm. These bands are assigned to the $\delta_{IP}{\left(C_{m}-Pyrrole\right)}_{x}+\tau \left(Pyridyl\right)$ and $\delta_{OP}\left(N-C_{\alpha }-C_{\beta }\right)$ vibrations, respectively, as seen in Table 1 and Table S2 in SI (illustrations 4 and 11). Although not present when the sample is excited at 488 nm, these bands are resonant with the 532 nm excitation at ambient conditions. The Raman features centered at $1007{\;cm}^{-1},$ $1150{\;cm}^{-1}$, and $1604\;cm^{-1}$ (Figure 16c,d), which are assigned to the $\nu \left(C_{\alpha }-C_{\beta }\right)$, $\delta_{IP}\left(N-H\right)$, and $\nu {\left(C-C\right)}_{Pyr}$ vibrations (Table 1 and Table S2 in SI (illustrations 24, 27, and 41)), present the same behavior: they appear in the spectrum obtained at 4.5 GPa with excitation at 488 nm, but they are not present when the pressure is set to 0.1 GPa. In addition, these bands are also resonant with the 532 nm excitation at ambient conditions. These results suggest that the resonance conditions of the porphyrin molecules are changing with changing pressure. In other words, the increase in pressure seems to result in an increased energy separation between electronic levels. Therefore, bands which are resonant at 532 nm (ambient conditions) become resonant at 488 nm for higher pressures. 

It is also important to note that the results associated with the molecules’ decompression show that the frequency shifts are reversible for most bands, but the vibrations between $970\;cm^{-1}$ and $1003\;cm^{-1}$ present signs of irreversibility (see Figure S3 in Supplementary Materials).

### 2.3. Low-Temperature Experiments

**Figure 17:**
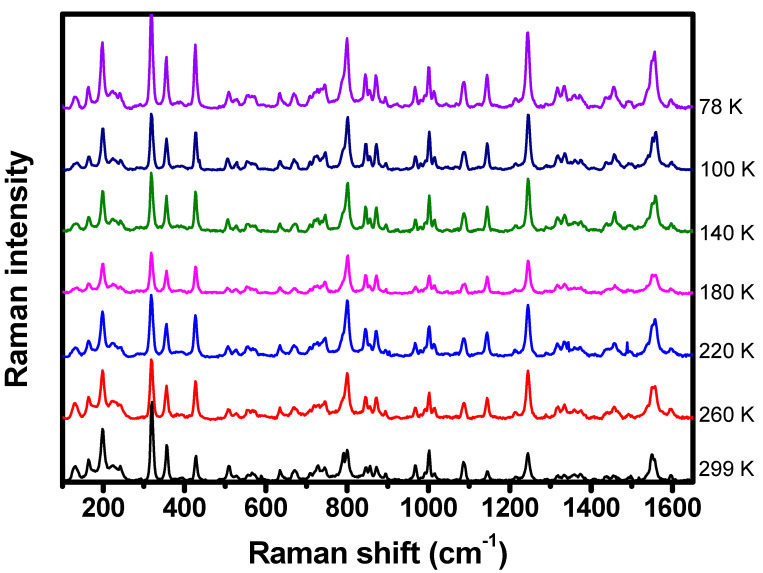
Raman spectrum of C-H_2_TPyP excited at 532 nm with temperatures ranging from 78 K to 299 K.

Temperature-dependent Raman spectroscopy has also been performed to complement the understand of porphyrin’s vibrational properties. The C-H_2_TPyP molecules were submitted to temperatures ranging from 78 K to 299 K. Differently from the behavior presented at variable pressures, no shifts in the Raman band centers or broadenings of the bands’ linewidths were detected in this range of temperatures, as shown in Figure 17. 

**Figure 18:**
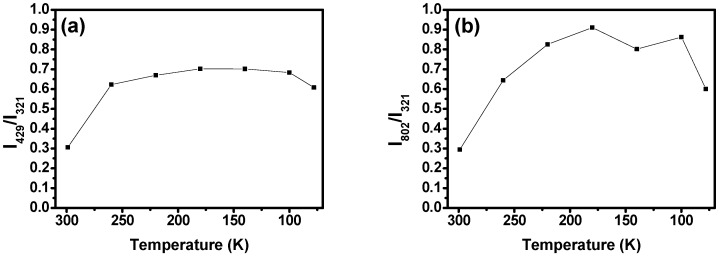
(**a**) $\tau_{IP}\left(Pyrrole\right)$ (at $429\;cm^{-1}$) and (**b**) $\delta {\left(C-H\right)}_{Pyr}$ (at $802\;cm^{-1}$) Raman modes’ relative intensities as a function of temperature. The relative intensities plotted here are the ratio of the modes’ absolute intensities with relation to the absolute intensity of the PBM mode ($321\;cm^{-1}$) at 299  K. The errors associated with the measurements of the relative intensities are lower than 0.1% for all acquired spectra.

The analysis of the relative intensities of the Raman bands with respect to the PBM ($321\;cm^{-1})$ intensity at 299  K shows that the intensities of most of the Raman modes remain essentially unchanged. However, some vibrations have their intensities greatly altered at lower temperatures, such as the $\tau_{IP}\left(Pyrrole\right)$ vibration at $429\;cm^{-1}$, whose relative intensity has an initial value of 0.3 at room temperature (299  K) that is increased to 0.7 at 180 K (see Figure 18a), and the $\delta {\left(C-H\right)}_{Pyr}$ mode at $801\;cm^{-1}$, whose relative intensity goes from 0.3 to 0.9 when the temperature is lowered from 299  K to 180  K; see Figure 18b. 

Overall, the effects observed in the relative intensities of the Raman bands are indicative of perturbations in the molecular symmetry at lower temperatures, which are likely altering the Raman activities of the modes. The graphs containing the temperature-dependent relative intensities for each assigned mode are available in Supplementary Materials; see Table S3.

Finally, the literature reports that isolated H_2_TPyP belongs to the $D_{2h}$ point group [37]. For this symmetry, it is expected that antisymmetric vibrational modes with respect to the molecule inversion center, called odd modes, will not be Raman-active [38,39]. Despite that, some vibrations observed in this work, at ambient conditions, higher pressures, and lower temperatures, are odd modes (for instance, $\tau_{OP}(Pyrrole)$ ($199\;{\;cm}^{-1}$) and $\delta {\left(C-C\right)}_{Pyr}$ ($367\;{\;cm}^{-1}$)). The observation of such modes in the Raman spectra strongly indicates that a reduction in the planarity of the molecule, and consequently a change in its symmetry, is taking place. We hypothesize that this planarity reduction could be associated with a saddle-shaped conformation (already observed in porphyrins [40]), due to local fields in the porphyrin crystal. Our theoretical predictions could only describe our experimental results after considering such symmetry change, predicting that H_2_TPyP assumes the $C_{2v}$ point group. In addition, the new vibration observed at $1175\;cm^{-1}$ at higher pressures is also an odd mode and possesses an antisymmetric vibration in the YZ plane. Its appearance indicates a further planarity modification with pressure, without further symmetry changes. Lastly, the changes in the relative intensities observed both at high pressures and low temperatures also indicate changes in the molecular symmetry.

## 3. Materials and Methods

C-H_2_TPyP was synthesized following the procedures described in reference [41], and the spectrometric analysis of the resulting crystals are in good agreement with the literature [42].

The vibrational properties of C-H_2_TPyP were investigated via Raman spectroscopy using a T64000 spectrometer from Horiba Jobin Yvon (Lille Country, France). The scattered light was collected using a 20× magnification objective lens in a backscattering configuration. The spectral resolution was $\pm 2{\;cm}^{-1}$. The measurements were conducted under both ambient conditions and high pressures. C-H_2_TPyP was excited using two different laser lines: 488 nm for both ambient and high-pressure conditions, and 532 nm for ambient conditions only. No fluorescence background was observed upon sample excitation at 488 nm. However, the issue of fluorescence background arises when the sample is excited with 532 nm. To address this problem, a baseline correction of the spectrum is performed. The baseline determination proceeds as follows: First, a numerical derivative of the experimental data is calculated. Since the fluorescence bands are generally much broader profiles compared to Raman bands, the first derivative is used to distinguish them. In the derivative spectrum, each Raman band appears as two symmetric bands around zero and the fluorescence signal grows as a background curve with the increase in the wavenumber. This fluorescence curve is then adjusted using a multiparametric function, integrated, and subsequently employed as the baseline for the original dataset.

The high-pressure Raman spectra were measured using a diamond anvil cell (μ-scope DAC HT(S)) from Almax easyLab (Diksmuider, Belgium). A mineral oil, specifically Nujol, was used as the pressure-transmitting medium [43]. The sample was loaded into a 100 μm hole drilled in a stainless-steel gasket (thickness of 200 μm), using an electric discharge machine from Almax easyLab. Pressure measurements were calibrated by monitoring the shifts in the ruby fluorescence lines [44,45]. The increase in fluorescence background originating from C-H_2_TPyP upon its insertion into the pressure cell makes it impractical to acquire the Raman signal using 532 nm excitation in high-pressure experiments.

The low-temperature Raman spectra measurements were performed with the Janis ST-500 cryostat from Lake shore Cryotornics (Westerville, OH, USA) and the samples, after being properly accommodated in the cryostat, were excited with a 532 nm (2.33 eV) CW laser using 40× objective lens with numerical aperture 0.60. The scattered light was acquired in a backscattering configuration, using an Andor SR303i spectrometer operating with a 1200 L/mm grating, coupled to an iDUS CCD camera. 

Our theoretical approach was based on the Density Functional Theory (DFT) formalism as implemented in the ORCA code [46], considering isolated molecules. We employed a polarized triple-zeta basis set (def2-TZVP) and the Generalized Gradient Approximation (GGA) within the Perdew–Burke–Ernzerhof (PBE) parametrization for the exchange–correlation functional. The calculated main bond lengths and angles are provided in supporting information (see Table S1 in Supplementary Materials) and were computed with both GGA (PBE) and META-GGA (M06-L). In Figure S2 in Supplementary Materials, the structure of H_2_TPyP computed with GGA (PBE) is used as reference to the analysis of Table S1 in Supplementary Materials. We found an excellent agreement between the two functionals, with the largest absolute difference in bond lengths being only 0.012 Å. Following the complete geometry optimization, the Raman spectra were determined numerically, with the best approximation to the experimental data achieved using the PBE functional, which is consistent with previous studies [47].

## 4. Conclusions

Raman-active vibrations in H_2_TPyP have been poorly explored and, in this work, through the combination of experiments and DFT calculations, we provide a thorough discussion of such vibrations. Every measured Raman-active vibration within $100{\;cm}^{-1}$ to $1700{\;cm}^{-1}$ that is resonant with either 532 nm or 488 nm is now assigned, with their symmetries and resonance properties properly addressed. In addition, the results show that the resonance conditions of active vibrations are tunable under hydrostatic pressure. In other words, bands which, under ambient conditions, are only active under 532 nm excitation become readily active at 488 nm with increasing pressure. Finally, H_2_TPyP has been reported to possess the point symmetry $D_{2h}$, but the experimental results presented here, combined with DFT calculations, suggest that these molecules are better described under the $C_{2v}$ symmetry. The pressure- and temperature-dependent results indicate that molecular planarity is being further perturbed at lower temperatures and higher pressures.

## Figures and Tables

**Table 1:** H_2_TPyP experimental and DFT–calculated Raman modes. In the table, ν stands for stretching; δ for bending; and τ for twist modes, respectively. The index “Pyr” identifies Raman modes related to the pyridyl ring. The indexes “IP” and “OP” stand for in-plane and out-of-plane modes, respectively. The indexes “x” and “y” indicate vibrations only in the respective direction.

Raman Mode	Symmetry (C_2v_)	Calculated (cm^−1^)	Experimental (cm^−1^)		
			488 nm Q_y_(0,2)	532 nm Q_y_(0,0)	
$\delta_{IP}\left(C_{m}-Pyrrole\right)$	$A_{1}$	163	164	160	Out-of-phase (XY) bending of the angles between the pyrrole groups.
$\tau_{OP}\left(Pyrrole\right)$	$A_{1}$	199	199	195	Out-of-phase twist of the pyrrole groups.
$\tau \left(Pyridyl\right)$	$A_{1}$	213	223	218	In-phase twist of the pyridyl groups.
$\delta_{IP}{\left(C_{m}-Pyrrole\right)}_{x}+\tau \left(Pyridyl\right)$	$B_{1}$	233	-	239	Bending of the angles between the $C_{m}$ and the X pyrrole groups and out-of-phase twist of the pyridyl groups.
PBM $\delta_{IP}\left(C_{m}-Pyrrole\right)$	$A_{1}$	327	321	317	Porphyrin Breathing Mode (PBM): In-phase bending of the angles between the pyrrole groups.
$\delta {\left(C-C\right)}_{Pyr}$	$A_{2}$	367	357	354	In-phase bending of the C−C bonds in pyridyl groups.
$\tau_{IP}\left(Pyrrole\right)$	$A_{2}$	427	-	426	Twist of the Y pyrrole groups.
$\delta {\left(C-C\right)}_{Pyr}+\delta {\left(C-N\right)}_{Pyr}$	$A_{1}$	501	-	511	Out-of-phase bending of the C−C and C−N bonds in the pyridyl groups.
$\delta_{OP}\left(C_{m}-C_{\alpha }-N\right)$	$A_{1}$	557	-	561	In-phase bending of the angles between the $C_{m}-C_{\alpha }$ and $C_{\alpha }-N$ bonds.
$\delta_{OP}\left(N-H\right)+\delta_{OP}\left(C_{\beta }-H\right)$	$A_{2}$	630	636	633	Out-of-phase bending of the N−H and $C_{\beta }-H$ bonds.
$\delta_{OP}\left(N-C_{\alpha }-C_{\beta }\right)$	$A_{2}$	672	-	666	In-phase bending of angles between the $N-C_{\alpha }$ and $C_{\alpha }-C_{\beta }$ bonds.
$\delta_{OP}\left(N-H\right)+\delta_{OP}\left(C_{\beta }-H\right)$	$A_{1}$	719	-	710	In-phase bending of the N−H and $C_{\beta }-H$ bonds.
$\delta_{OP}\left(C_{m}-C_{\alpha }-C_{\beta }\right)$	$A_{2}$	739	-	730	Out-of-phase bending of angles between the $C_{m}-C_{\alpha }$ and $C_{\alpha }-C_{\beta }$ bonds.
$\delta {\left(C-H\right)}_{Pyr}$	$A_{2}$	752	-	744	In-phase bending of the C−H bonds in the pyridyl groups.
$\delta_{OP}\left(C_{\beta }-H\right)$	$A_{1}$	772	-	786	Out-of-phase bending of the $C_{\beta }-H$ bonds.
$\delta {\left(C-H\right)}_{Pyr}$	$A_{2}$	789	-	797	In-phase bending of the C−H bonds in the pyridyl groups.
$\delta {\left(C-H\right)}_{Pyr}$	$A_{2}$	859	-	844	In-phase bending of the C−H bonds in the pyridyl groups.
$\delta {\left(C-H\right)}_{Pyr}$	$A_{1}$	864	-	855	Out-of-phase bending of the C−H bonds in the pyridyl groups.
$\delta_{OP}(C_{\beta }-H{)}_{y}$	$A_{2}$	884	-	871	Bending of the $C_{\beta }-H$ in the Y pyrrole groups.
$\delta_{IP}\left(C_{m}-C_{\alpha }-N\right)$	$A_{1}$	887	-	892	In-phase bending of the angles between the $C_{m}-C_{\alpha }$ and $N-C_{\alpha }$ bonds.
$\delta {\left(C-H\right)}_{Pyr}$	$A_{1}$	966	967	966	Out-of-phase bending of the C−H bonds in the pyridyl groups.
$\delta {\left(C-N\right)}_{Pyr}+\delta {\left(C-C\right)}_{Pyr}$	$A_{1}$	980	991	989	In-phase bending of the C−N and C−C bonds.
$\delta_{IP}{\left(C_{\beta }-H\right)}_{x}+\nu {\left(N-C_{\alpha }-C_{\beta }\right)}_{x}$	$A_{2}$	1003	1001	1000	Bending of the $C_{\beta }-H$ bonds and stretching of the $N-C_{\alpha }-C_{\beta }$ bonds in the X pyrrole groups.
$\nu \left(C_{\alpha }-C_{\beta }\right)$	$A_{1}$	1006	1017	1014	Out-of-phase stretching of the $C_{\alpha }-C_{\beta }$ bonds.
$\delta_{IP}\left(C_{\beta }-H\right)$	$A_{1}$	1065	1063	1063	In-phase bending of the $C_{\beta }-H$ bonds.
$\delta_{IP}\left(C_{\beta }-H\right)$	$A_{1}$	1069	1086	1085	Out-of-phase bending of the $C_{\beta }-H$ bonds.
$\delta_{IP}\left(N-H\right)$	$A_{2}$	1122	-	1142	Bending of the N−H bonds.
$\delta {\left(C-H\right)}_{Pyr}$	$A_{1}$	1206	1211	1213	Out-of-phase bending of the C−H bonds in the pyridyl groups.
$\nu \left(C_{m}-Pyridyl\right)+\delta {\left(C-H\right)}_{Pyr}$	$A_{1}$	1235	1241	1241	In-phase stretching of the $C_{m}-Pyridyl$ bonds and bending of the C−H bonds in the pyridyl groups.
$\delta_{IP}{\left(N-C_{\alpha }\right)}_{x}+\nu {\left(C_{\alpha }-C_{\beta }\right)}_{x}+\nu {\left(N-C_{\alpha }\right)}_{y}$	$A_{1}$	1289	1287	-	Bending of the $N-C_{\alpha }$ bonds and stretching of the $C_{\alpha }-C_{\beta }$ bonds in the X pyrrole groups. Stretching of the $N-C_{\alpha }$ bonds in the Y pyrrole groups.
$\delta {\left(C-H\right)}_{Pyr}$	$A_{2}$	1310	1324	1314	Out-of-phase bending of the C−H bonds in the pyridyl groups.
$\delta_{IP}\left(C_{\beta }-H\right)+\nu {\left(C_{\alpha }-C_{\beta }\right)}_{x}$	$A_{2}$	1316	1324	1330	In-phase bending of the $C_{\beta }-H$ bonds. Stretching of the $C_{\alpha }-C_{\beta }$ in the X pyrrole groups
$\delta_{IP}\left(N-C_{\alpha }\right)+\nu \left(C_{\alpha }-C_{\beta }\right)$	$A_{1}$	1356	1357	1357	In-phase bending of the angles between the $N-C_{\alpha }$ bonds and stretching of the $C_{\alpha }-C_{\beta }$ bonds.
$\delta_{IP}\left(C_{\beta }-H\right)+\nu {\left(N-C_{\alpha }-C_{\beta }\right)}_{y}$	$A_{2}$	1366	1373	1373	Bending of the $C_{\beta }-H$ bonds. Stretching of the $N-C_{\alpha }$ and $C_{\alpha }-C_{\beta }$ bonds in the Y pyrrole groups.
$\nu \left(C_{\beta }-C_{\beta }\right)+\nu \left(C_{m}-C_{\alpha }-N\right)$	$A_{1}$	1438	1436	1434	In-phase stretching of the $C_{\beta }-C_{\beta }$, $C_{m}-C_{\alpha }$, and $N-C_{\alpha }$ bonds.
$\nu {\left(C_{m}-C_{\alpha }\right)}_{x}+\nu {\left(C_{\alpha }-C_{\beta }\right)}_{x}$	$A_{2}$	1448	1454	1451	Stretching of the $C_{m}-C_{\alpha }$ and $C_{\alpha }-C_{\beta }$ bonds in the X pyrrole groups.
$\delta {\left(C-H\right)}_{Pyr}$	$A_{2}$	1474	1470	-	Out-of-phase bending of the C−H bonds in the pyridyl groups.
$\nu \left(C_{\beta }-C_{\beta }\right)+\nu {\left(C_{m}-C_{\alpha }-N\right)}_{y}$	$A_{1}$	1499	1489	1495	Out-of-phase stretching of the $C_{\beta }-C_{\beta }$. Stretching of the $C_{m}-C_{\alpha }$ and $N-C_{\alpha }$ bonds in the Y pyrrole groups.
$\nu \left(C_{\beta }-C_{\beta }\right)+\nu {\left(C_{m}-C_{\alpha }-N\right)}_{x}$	$A_{1}$	1545	1538	-	In-phase stretching of the $C_{\beta }-C_{\beta }$ bonds. Stretching of the $C_{m}-C_{\alpha }$ and $N-C_{\alpha }$ bonds in the X pyrrole groups.
$\delta_{IP}{\left(N-C_{\alpha }\right)}_{y}+\nu {\left(C_{\beta }-C_{\beta }\right)}_{x}+\nu \left(C_{m}-C_{\alpha }\right)$	$A_{1}$	1554	1553	1549	Bending of the angles between the $N-C_{\alpha }$ bonds in the Y pyrrole groups. Stretching of the $C_{\beta }-C_{\beta }$ bonds in the X pyrrole groups. Out-of-phase stretching of the $C_{m}-C_{\alpha }$ bonds.
$\nu {\left(C-C\right)}_{Pyr}$	$A_{1}$	1581	1589	1580	In-phase stretching of the C−C bonds in the pyridyl groups.

**Table 2:** Experimental dω/dP rates for the observed Raman bands. CLV stands for Crystal Lattice Vibration. Some Raman modes present two slopes with pressure; their intercept and dω/dP at such pressures are indicated as follows: ^#^ 0.8 GPa, ** 1.5 Gpa, * 2.5 Gpa, and ^$^ 5.6 GPa. The numbers in brackets are the errors in the intercept and dω/dP rates obtained from fitting.

Raman Mode	Intercept Position at 0.1 GPa (cm^−1^)	dω/dP (cm^−1^/GPa)
CLV	81.2 (3.6); 100.4 (2.6) *	10.1 (2.0); 1.6 (0.8) *
CLV	98.5 (1.4)	10.1 (2.7)
CLV	102.6 (0.9); 117.6 (1.4) **	14.6 (0.9); 5.6 (0.3) **
CLV	116.6 (1.0)	14.7 (1.1)
CLV	133.2 (2.2)	25.0 (4.3)
$\delta_{IP}\left(C_{m}-Pyrrole\right)$	162.9 (1.1)	8.1 (2.1)
$\tau_{OP}\left(Pyrrole\right)$	203.1 (0.8)	3.8 (0.2)
$\tau \left(Pyridyl\right)$	221.4 (0.4)	2.1 (0.6)
$\delta_{IP}\left(C_{m}-Pyrrole\right)+\tau \left(Pyridyl\right)$	242.0 (1.7)	10.1 (3.2)
PBM	322.8 (0.4)	2.7 (0.1)
$\delta {\left(C-C\right)}_{Pyr}$	358.9 (0.5)	3.1 (0.2)
$\delta_{OP}\left(N-H\right)+\delta_{OP}\left(C_{\beta }-H\right)$	636.1 (0.5)	1.3 (0.1)
$\delta \left(N-C_{\alpha }-C_{\beta }\right)$	642.0 (1.2); 670.0 (0.8) *	3.3 (1.1); 1.3 (0.2) *
$\delta {\left(C-H\right)}_{Pyr}$	970.4 (0.4); 1017.6 (5.0) ^$^	2.3 (0.1); −1.1 (0.7) ^$^
$\delta {\left(C-N\right)}_{Pyr}+\delta {\left(C-C\right)}_{Pyr}$	993.8 (0.7)	2.3 (0.2)
$\delta_{IP}{\left(C_{\beta }-H\right)}_{x}+\nu {\left(N-C_{\alpha }-C_{\beta }\right)}_{x}$	1004.1 (0.7)	2.9 (0.2)
$\delta_{IP}\left(C_{\beta }-H\right)$	1066.4 (0.8)	3.3 (0.2)
$\delta_{IP}\left(C_{\beta }-H\right)$	1088.0 (0.5)	3.5 (0.1)
$\delta_{IP}\left(N-H\right)$	1145.9 (0.8); 1142.7 (0.8) **	−8.8 (1.2); 2.5 (0.2) **
$\delta_{IP}\left(N-H\right)$	1167.5 (1.0) *	3.0 (0.2) *
$\delta {\left(C-H\right)}_{Pyr}$	1216.8 (0.5)	2.5 (0.1)
$\nu \left(C_{m}-Pyridyl\right)+\delta {\left(C-H\right)}_{Pyr}$	1250.5 (0.9)	4.2 (0.2)
$\nu \left(C_{\beta }-C_{\beta }\right)+\nu \left(C_{m}-C_{\alpha }-N\right)$	1438.7 (0.4)	2.6 (0.1)
$\nu {\left(C_{m}-C_{\alpha }\right)}_{x}+\nu {\left(C_{\alpha }-C_{\beta }\right)}_{x}$	1455.8 (0.5)	4.0 (0.1)
$\nu \left(C_{\beta }-C_{\beta }\right)+\nu {\left(C_{m}-C_{\alpha }-N\right)}_{y}$	1488.6 (1.1)	11.7 (2.1)
$\nu \left(C_{\beta }-C_{\beta }\right)+\nu {\left(C_{m}-C_{\alpha }-N\right)}_{x}$	1537.4 (0.3); 1550.8 (0.4) ^#^	11.4 (0.7); 2.9 (0.1) ^#^
$\delta_{IP}{\left(N-C_{\alpha }\right)}_{y}+\nu {\left(C_{\beta }-C_{\beta }\right)}_{x}+\nu \left(C_{m}-C_{\alpha }\right)$	1555.5 (0.7); 1563.5 (0.6) ^#^	7.5 (1.6); 4.5(0.1) ^#^
$\nu {\left(C-C\right)}_{Pyr}$	1602.9 (0.8)	2.5 (0.2)

## Data Availability

Data are contained within the article and Supplementary Materials.

## Supplementary Materials

The following supporting information can be downloaded at: https://www.mdpi.com/article/10.3390/molecules29102362/s1, Figure S1: X-ray diffractogram of C-H_2_TPyP. The peaks reveal a crystalline structure of the investigated sample. Figure S2: Structure of H_2_TPyP molecule calculated with GGA (PBE) and used as reference to Table S2 in SI. Figure S3: Raman shift of the C-H_2_TPyP bands under compression (▲) and decompression (∇). Table S1: Main bond lengths determined by DFT calculations employing two choices of exchange-correlation functionals, GGA (PBE) and META-GGA (M06-L). The bonds are indicated in Figure S2, which presents the structure of optimized porphyrin. The two functionals are in excellent agreement with each other: the largest absolute difference is only 0.012 Å, while the largest percentage difference is 0.9%. Table S2: Schematic illustrations of the Raman modes observed in this work. The black and red lines indicate out-of-plane opposite bonds. The arrows are in-plane vibrations, and the symbols ⊙ and ⊗ are out-of-plane opposite vibrations. Green and purple arrows represent out-of-phase modes. The indexes “IP” and “OP” stand for in-plane and out-of-plane modes, respectively. The indexes “x” and “y” indicate vibrations only in the respective direction. Table S3: Raman relative intensity (Y-axis) of C-H_2_TPyP bands, relative to the PBM mode at 321 cm^−1^, as function of temperature (X-axis), ranging from 299 K to 76 K. The inserted values indicate the ratio between the intensity at the analyzed Raman band frequency (in cm^−1^) and the reference (PBM).
