# Supplementary material for: Raman Spectroscopy on Free-Base Meso-tetra(4-pyridyl) Porphyrin under Conditions of Low Temperature and High Hydrostatic Pressure
Source: Molecules. 2024 May 17;29(10):2362. doi: 10.3390/molecules29102362 (PMC11124280; doi:10.3390/molecules29102362)
Supplement: Supplementary file 1 [file molecules-29-02362-s001.zip › molecules-2890547-supplementary.pdf]

Supplementary Materials

Article

Raman Spectroscopy on Free-Base Meso-tetra(4-pyridyl) Porphyrin under Conditions of Low Temperature and High Hydrostatic Pressure

Jhon Rewllyson Torres dos Reis [1], Fabio Furtado Leite [1,2], Keshav Sharma [3], Guilherme Almeida Silva Ribeiro [4], Welesson Henrique Natanal Silva [4], Alzir Azevedo Batista [5], Alexandre Rocha Paschoal [6], Waldeci Paraguassu [1], Mario Mazzoni [4], Newton Martins Barbosa Neto [1,*] and Paulo Trindade Araujo [3,*]

[1] Graduate Program in Physics, Institute of Natural Sciences, Federal University of Pará, Belém 66075-110, PA, Brazil; jhon.rewllyson@gmail.com (J.R.T.d.R.); fabioleite@unifap.br (F.F.L.); paraguassu@ufpa.br (W.P.)
[2] Department of Exact and Technological Sciences, Federal University of Amapá, Macapá 68903-419, AP, Brazil
[3] Department of Physics and Astronomy, University of Alabama, Tuscaloosa, AL 35487, USA; ksharma1@crimson.ua.edu
[4] Department of Physics, Federal University of Minas Gerais, Belo Horizonte 31270-901, MG, Brazil; almeida.guilherme13@gmail.com (G.A.S.R.); welessonhenrique@gmail.com (W.H.N.S.); mmazzonibh@gmail.com (M.M.)
[5] Departament of Chemistry, Federal University of São Carlos, São Carlos 13565-905, SP, Brazil; daab@ufscar.br
[6] Department of Physics, Federal University of Ceara, Fortaleza 60455-760, CE, Brazil; paschoal@fisica.ufc.br
* Correspondence: barbosaneto@ufpa.br (N.M.B.N.); paulo.t.araujo@ua.edu (P.T.A.)

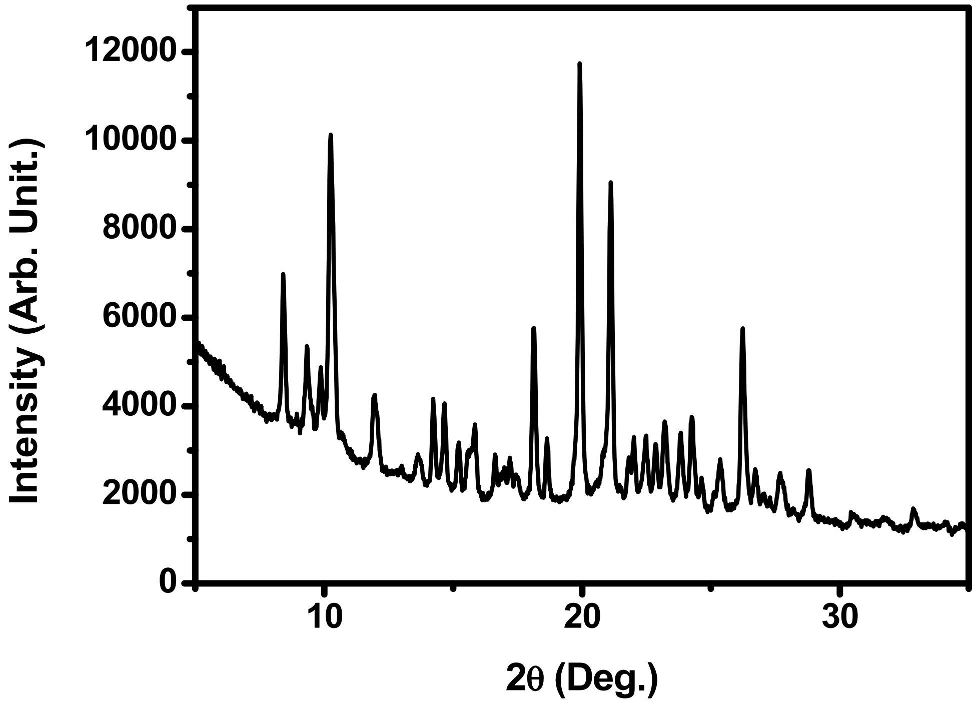

Figure S1: X-ray diffractogram of C-H_2TPyP. The peaks reveal a crystalline structure of the investigated sample.

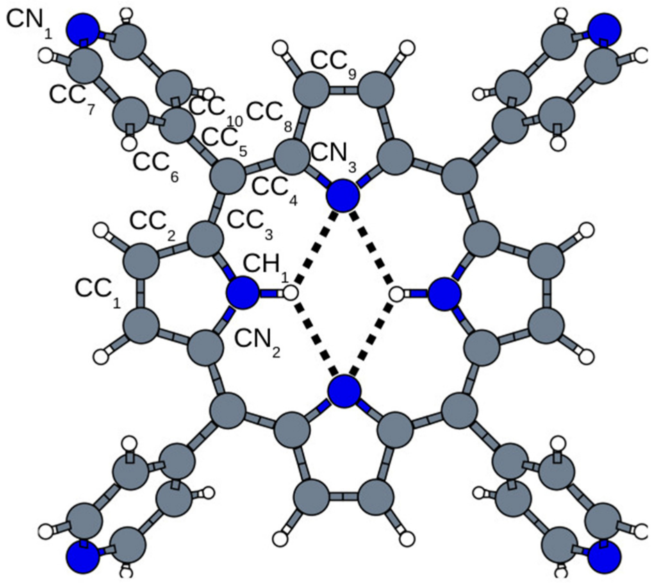

Figure S2: Structure of H_2TPyP molecule calculated with GGA (PBE) and used as reference to **Table S2 in SI**.

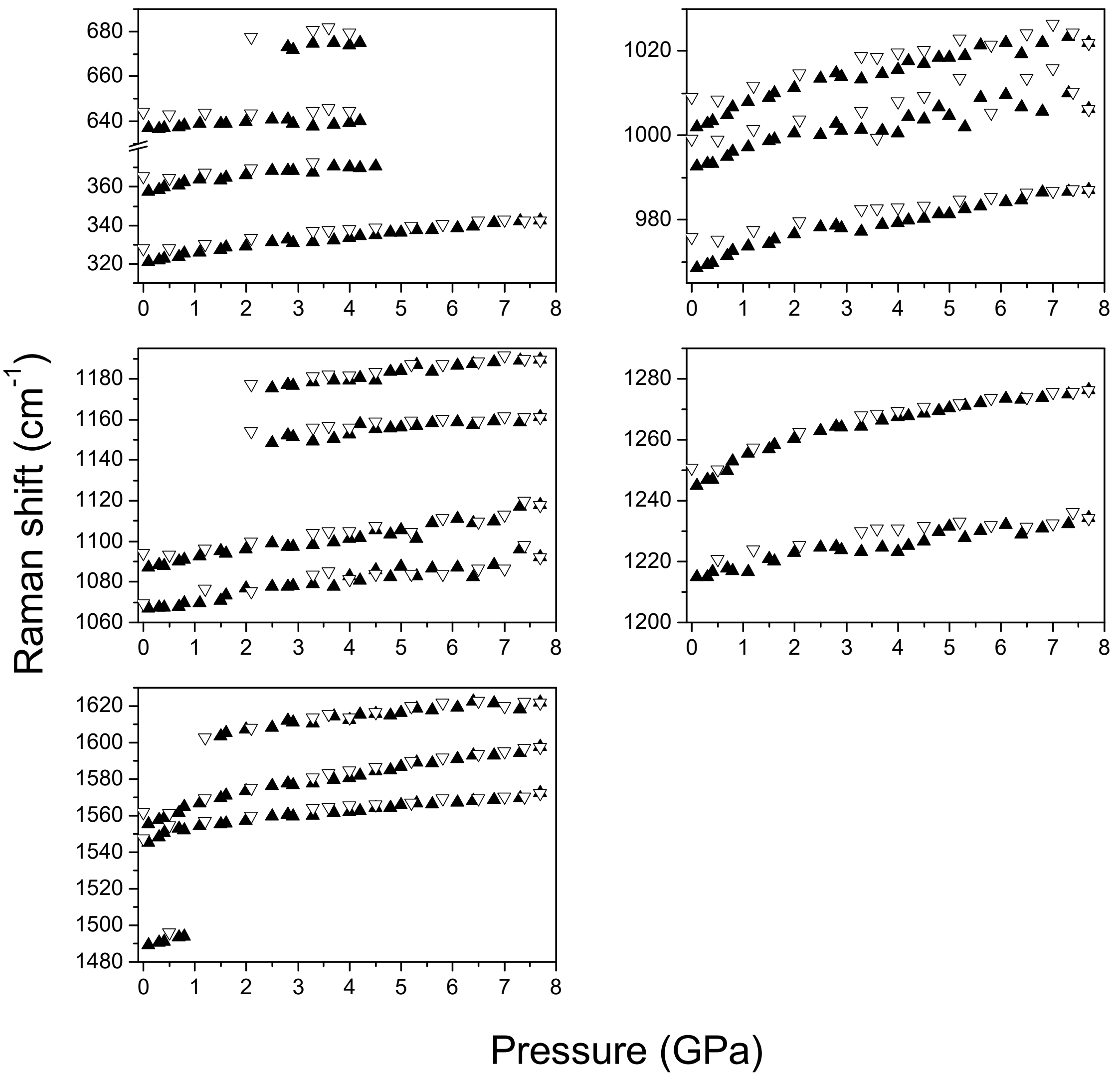

Figure S3: Raman shift of the C-H_2TPyP bands under compression (▲) and decompression (∇).

Table S1: Main bond lengths determined by DFT calculations employing two choices of exchange-correlation functionals, GGA (PBE) and META-GGA (M06-L). The bonds are indicated in **Figure S2**, which presents the structure of optimized porphyrin. The two functionals are in excellent agreement with each other: the largest absolute difference is only 0.012 Å, while the largest percentage difference is 0.9 %.

**Bond**	**GGA:PBE (Å)**	**MET-GGA:M06-L (Å)**
CC1	1.373	1.362
CC2	1.434	1.422
CC3	1.407	1.398
CC4	1.413	1.405
CC5	1.492	1.481
CC6	1.402	1.394
CC7	1.396	1.385
CC8	1.458	1.447
CC9	1.358	1.347
CN1	1.341	1.331
CN2	1.376	1.368
CN3	1.370	1.360
NH1	1.021	1.012

The angles C-N(H)-C and C-N-C involving the central nitrogen atoms are 110.6^0 and 105.3^0 (GGA) and 110.5^0 and 105.1^0 (META-GGA), respectively. The torsion angle (following the four atoms involved in the bonds CC4-CC5-CC10 are 65^0 and 58^0 for GGA and META-GGA, respectively.

Table S2: Schematic illustrations of the Raman modes observed in this work. The black and red lines indicate out-of-plane opposite bonds. The arrows are in-plane vibrations, and the symbols ⊙ and ⊗ are out-of-plane opposite vibrations. Green and purple arrows represent out-of-phase modes. The indexes “IP” and “OP” stand for in-plane and out-of-plane modes, respectively. The indexes “x” and “y” indicate vibrations only in the respective direction.

(1)<br>$\delta_{IP}(C_m - Pyrrole)$<br>$163\ cm^{-1}$<br>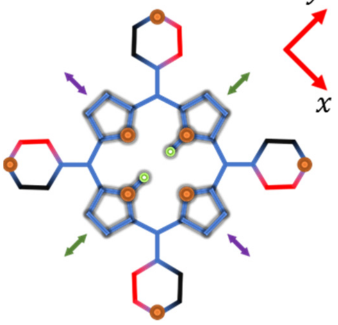	(2)<br>$\tau_{OP}(Pyrrole)$<br>$199\ cm^{-1}$<br>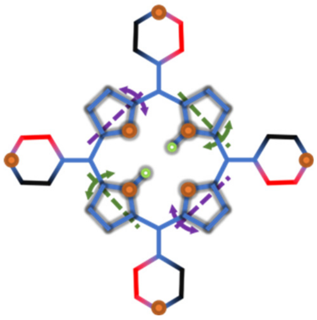	(3)<br>$\tau(Pyridyl)$<br>$213\ cm^{-1}$<br>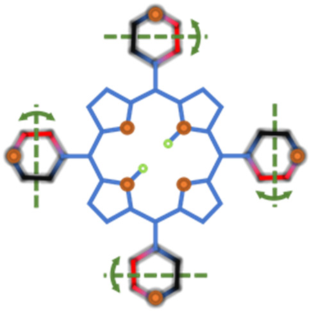
(4)<br>$\delta_{IP}(C_m - Pyrrole)_x$<br>$+\tau(Pyridyl)$<br>$233\ cm^{-1}$<br>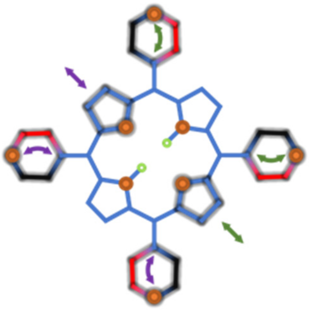	(5)<br>**PBM**<br>$\delta_{IP}(C_m - Pyrrole)$<br>$327\ cm^{-1}$<br>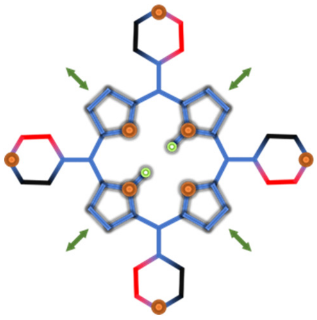	(6)<br>$\delta(C - C)_{Pyr}$<br>$367\ cm^{-1}$<br>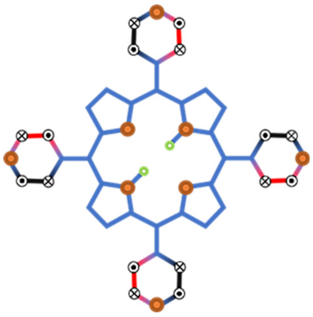

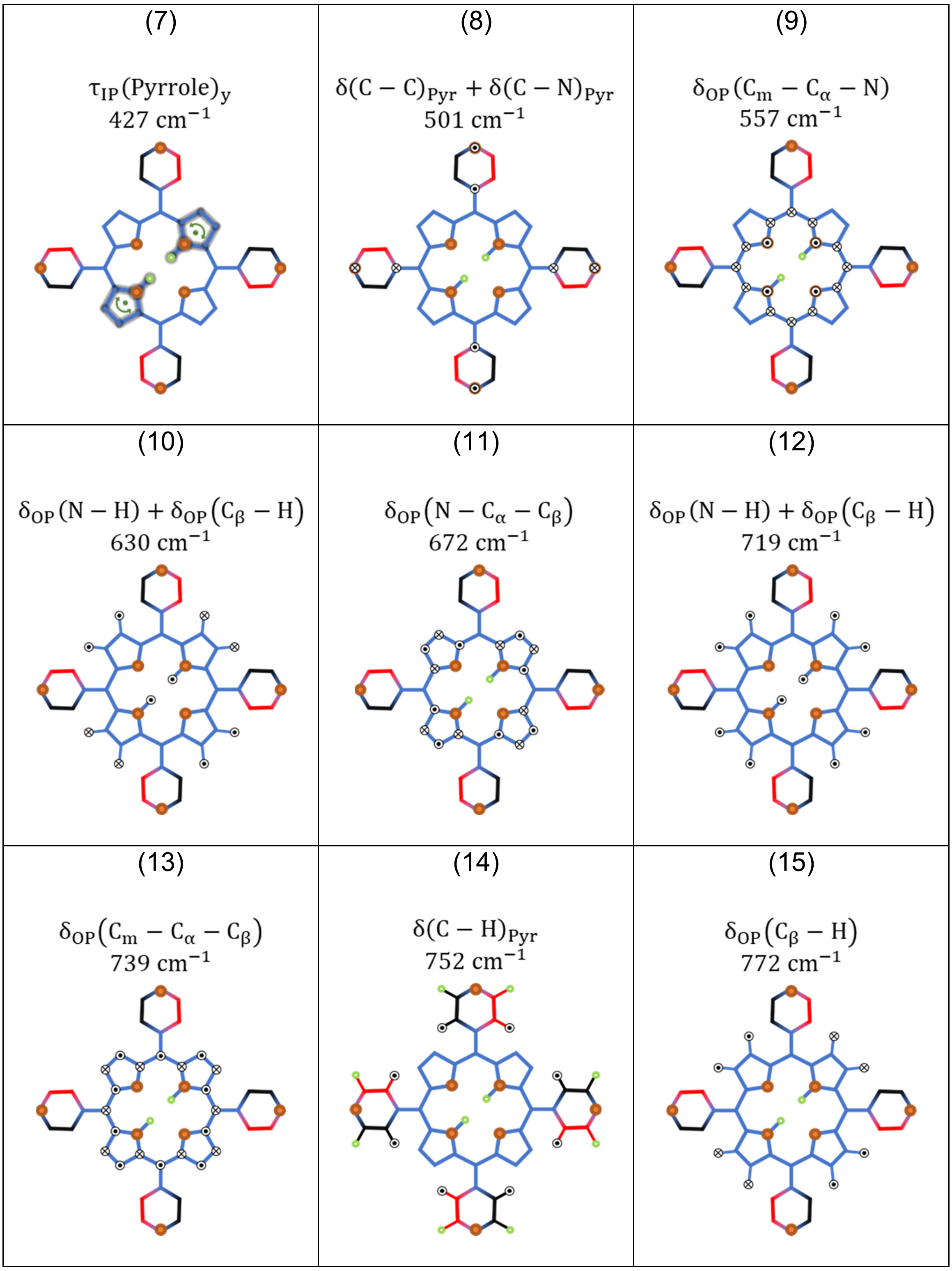
(7)
$\tau_{IP}(\mathrm{Pyrrole})_y$
427 cm^{-1}
(8)
$\delta(C-C)_{Pyr} + \delta(C-N)_{Pyr}$
501 cm^{-1}
(9)
$\delta_{OP}(C_m - C_\alpha - N)$
557 cm^{-1}
(10)
$\delta_{OP}(N-H) + \delta_{OP}(C_\beta - H)$
630 cm^{-1}
(11)
$\delta_{OP}(N - C_\alpha - C_\beta)$
672 cm^{-1}
(12)
$\delta_{OP}(N-H) + \delta_{OP}(C_\beta - H)$
719 cm^{-1}
(13)
$\delta_{OP}(C_m - C_\alpha - C_\beta)$
739 cm^{-1}
(14)
$\delta(C-H)_{Pyr}$
752 cm^{-1}
(15)
$\delta_{OP}(C_\beta - H)$
772 cm^{-1}

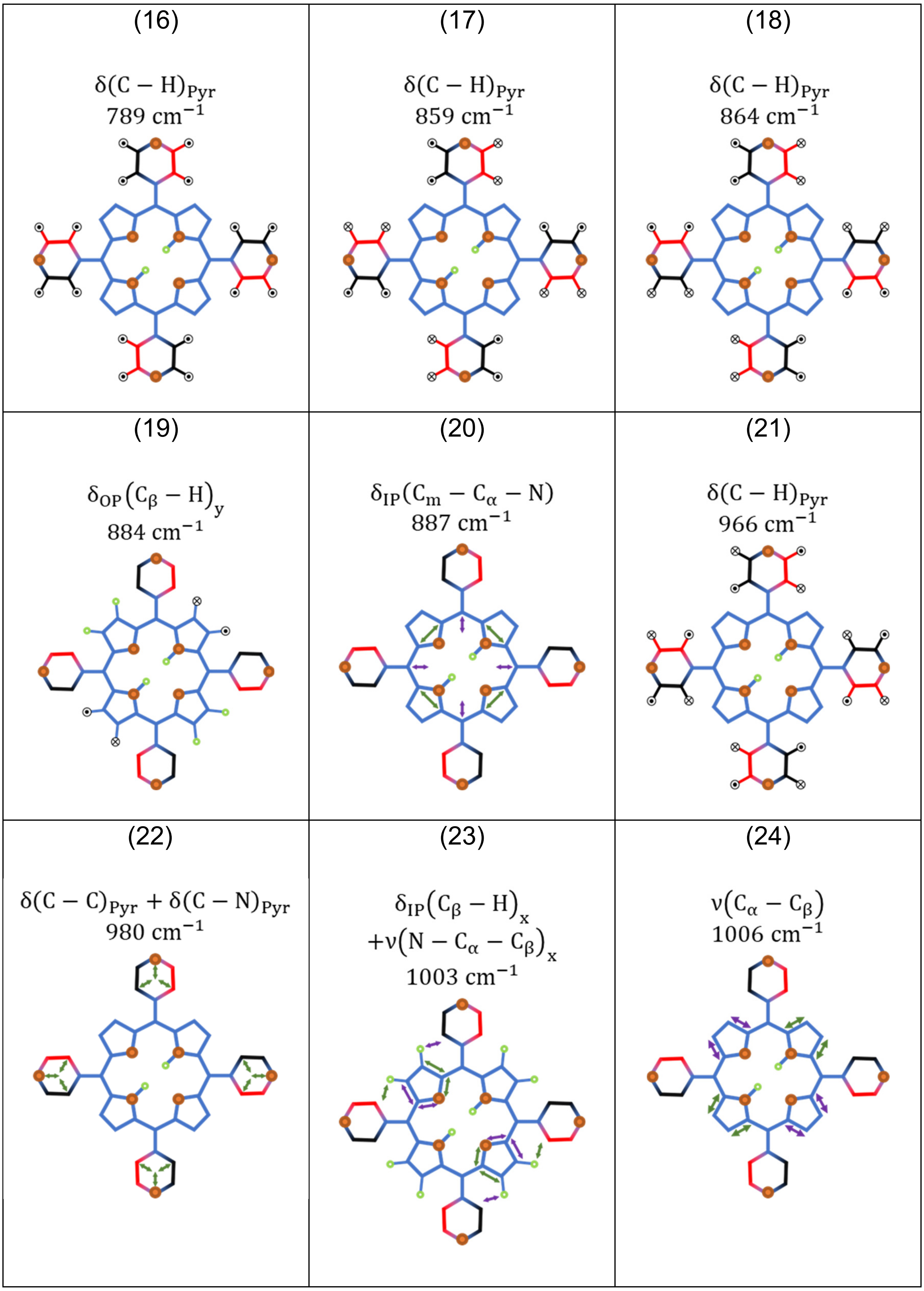
(16)
$\delta(C-H)_{Pyr}$
$789\ cm^{-1}$
(17)
$\delta(C-H)_{Pyr}$
$859\ cm^{-1}$
(18)
$\delta(C-H)_{Pyr}$
$864\ cm^{-1}$
(19)
$\delta_{OP}(C_\beta-H)_y$
$884\ cm^{-1}$
(20)
$\delta_{IP}(C_m-C_\alpha-N)$
$887\ cm^{-1}$
(21)
$\delta(C-H)_{Pyr}$
$966\ cm^{-1}$
(22)
$\delta(C-C)_{Pyr}+\delta(C-N)_{Pyr}$
$980\ cm^{-1}$
(23)
$\delta_{IP}(C_\beta-H)_x$
$+\nu(N-C_\alpha-C_\beta)_x$
$1003\ cm^{-1}$
(24)
$\nu(C_\alpha-C_\beta)$
$1006\ cm^{-1}$

(25)<br>$\delta_{IP}(C_\beta - H)$<br>$1065\ cm^{-1}$<br>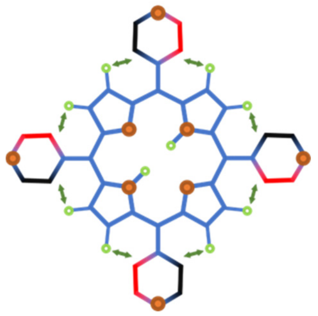	(26)<br>$\delta_{IP}(C_\beta - H)$<br>$1069\ cm^{-1}$<br>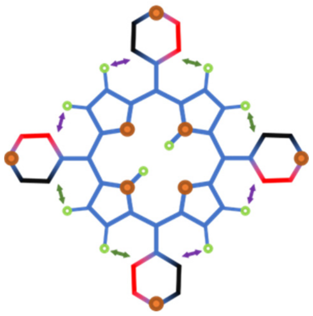	(27)<br>$\delta_{IP}(N - H)$<br>$1122\ cm^{-1}$<br>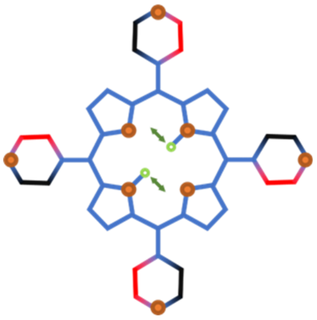
(28)<br>$\delta(C - H)_{Pyr}$<br>$1206\ cm^{-1}$<br>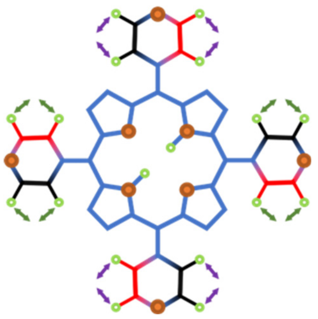	(29)<br>$\nu(C_m - Pyridyl)$<br>$+\delta(C - H)_{Pyr}$<br>$1235\ cm^{-1}$<br>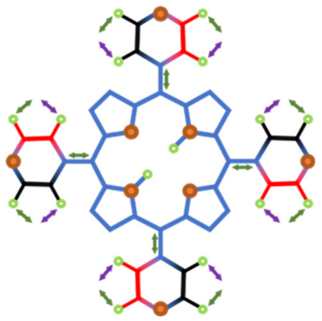	(30)<br>$\delta_{IP}(N - C_\alpha)_x + \nu(C_\alpha - C_\beta)_x$<br>$+\nu(N - C_\alpha)_y$<br>$1289\ cm^{-1}$<br>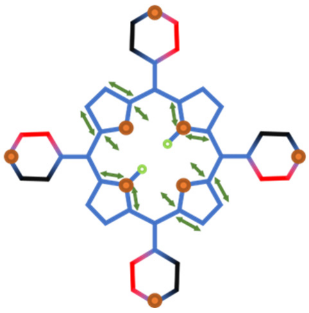
(31)<br>$\delta(C - H)_{Pyr}$<br>$1310\ cm^{-1}$<br>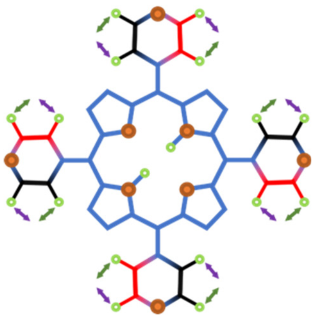	(32)<br>$\delta_{IP}(C_\beta - H)$<br>$+\nu(C_\alpha - C_\beta)_x$<br>$1316\ cm^{-1}$<br>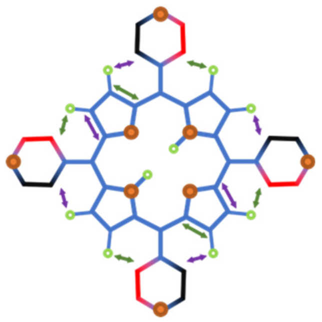	(33)<br>$\delta_{IP}(N - C_\alpha) + \nu(C_\alpha - C_\beta)$<br>$1356\ cm^{-1}$<br>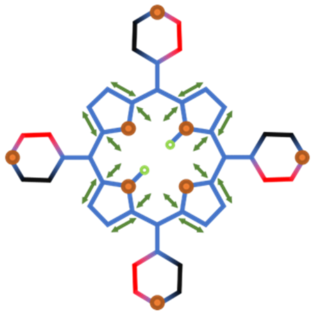

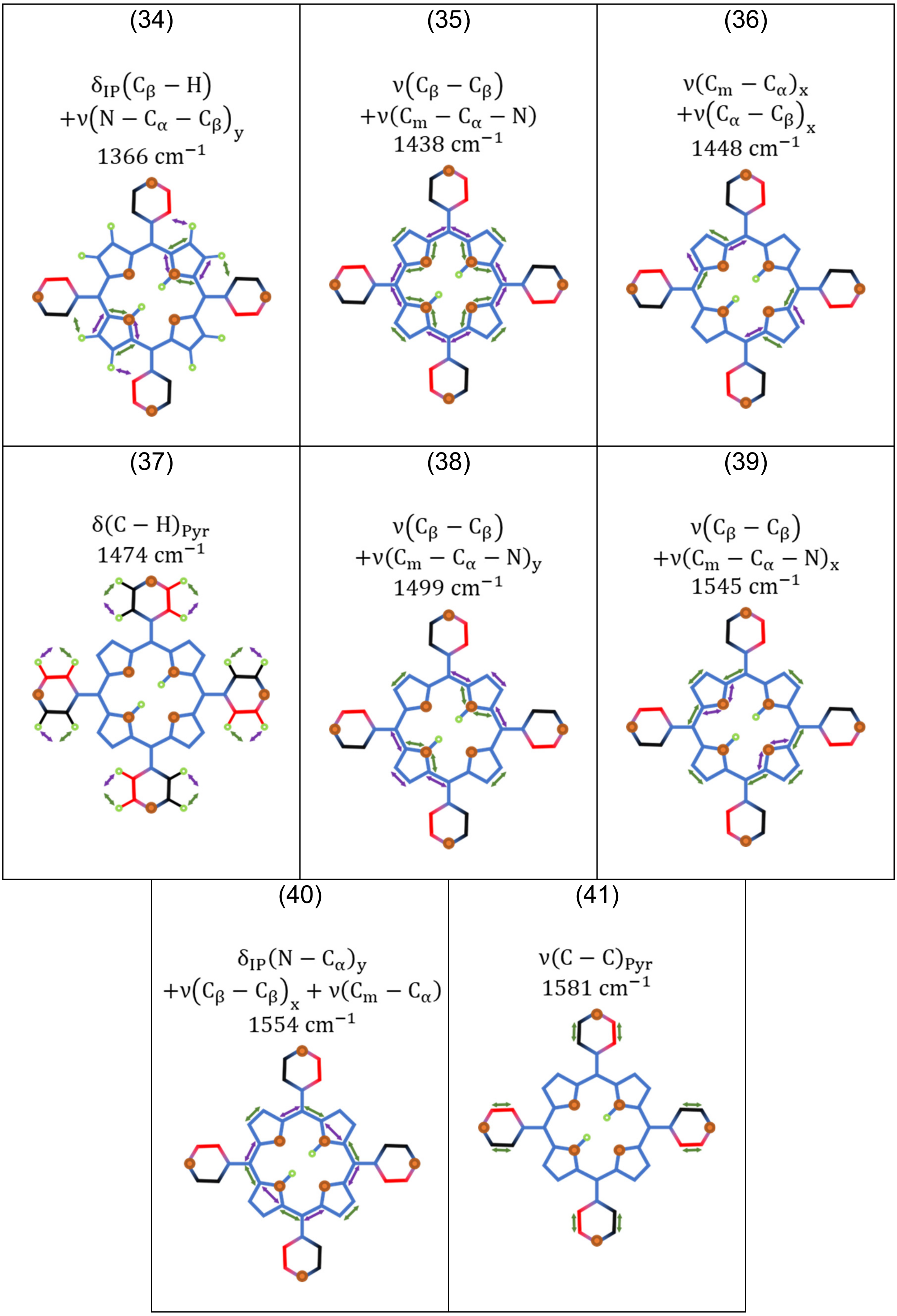
(34)
$\delta_{IP}(C_\beta - H)$
$+\nu(N - C_\alpha - C_\beta)_y$
$1366\ cm^{-1}$
(35)
$\nu(C_\beta - C_\beta)$
$+\nu(C_m - C_\alpha - N)$
$1438\ cm^{-1}$
(36)
$\nu(C_m - C_\alpha)_x$
$+\nu(C_\alpha - C_\beta)_x$
$1448\ cm^{-1}$
(37)
$\delta(C - H)_{Pyr}$
$1474\ cm^{-1}$
(38)
$\nu(C_\beta - C_\beta)$
$+\nu(C_m - C_\alpha - N)_y$
$1499\ cm^{-1}$
(39)
$\nu(C_\beta - C_\beta)$
$+\nu(C_m - C_\alpha - N)_x$
$1545\ cm^{-1}$
(40)
$\delta_{IP}(N - C_\alpha)_y$
$+\nu(C_\beta - C_\beta)_x + \nu(C_m - C_\alpha)$
$1554\ cm^{-1}$
(41)
$\nu(C - C)_{Pyr}$
$1581\ cm^{-1}$

Table S3: Raman relative intensity (Y-axis) of C-H_2TPyP bands, relative to the PBM mode at $321\ \mathrm{cm}^{-1}$, as function of temperature (X-axis), ranging from $299\ \mathrm{K}$ to $76\ \mathrm{K}$. The inserted values indicate the ratio between the intensity at the analyzed Raman band frequency (in cm^{-1}) and the reference (PBM).

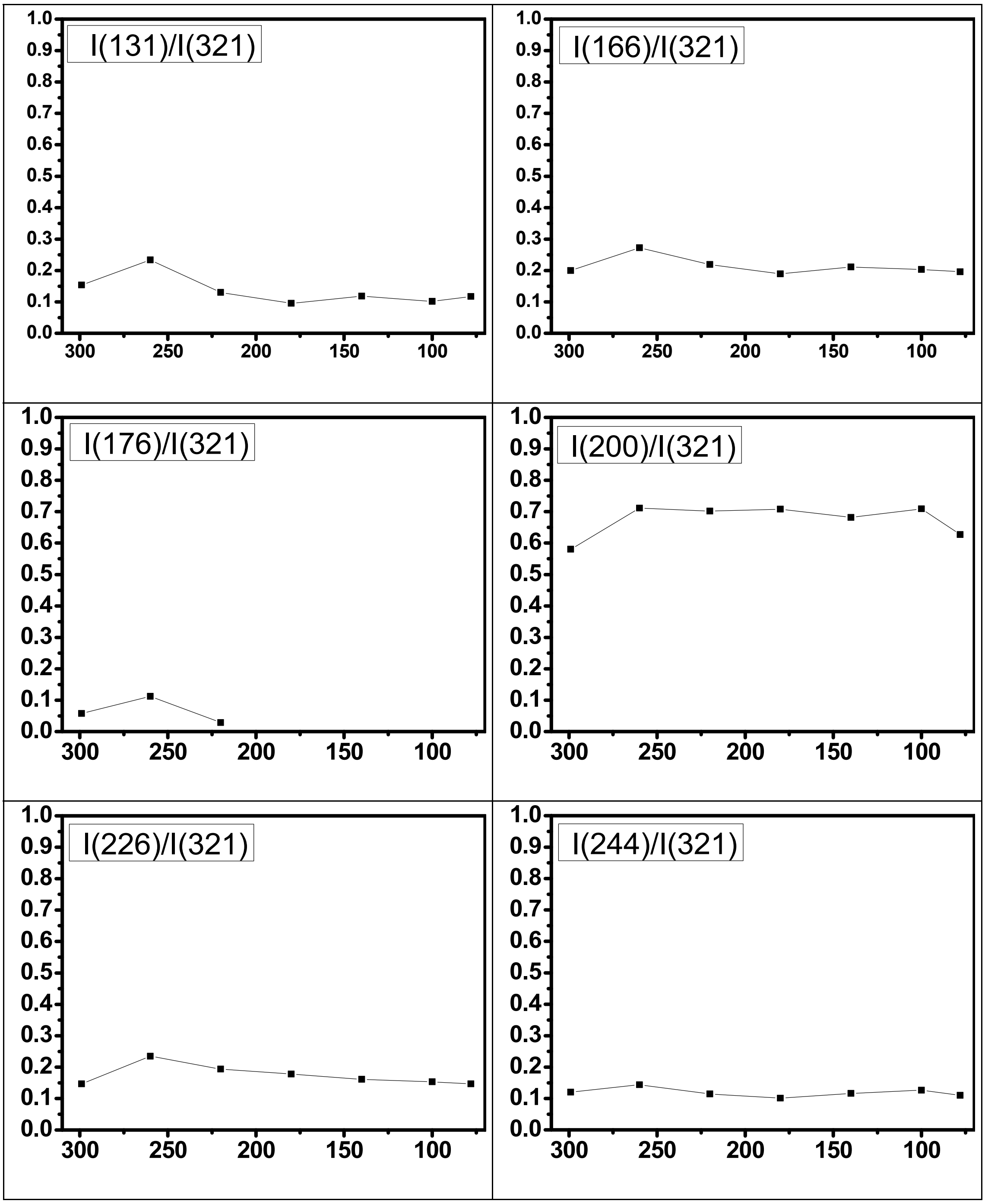

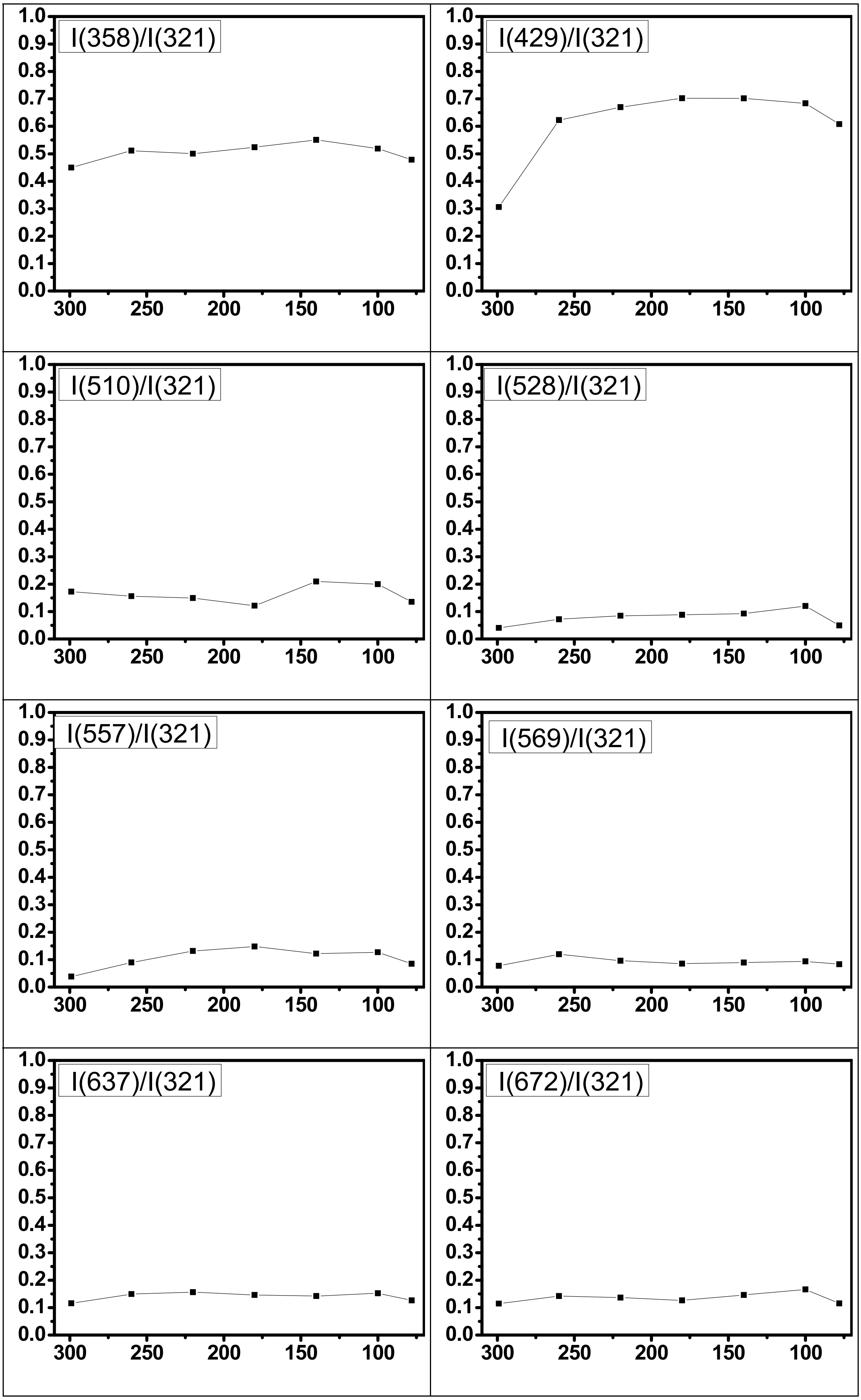
I(358)/I(321)
I(429)/I(321)
I(510)/I(321)
I(528)/I(321)
I(557)/I(321)
I(569)/I(321)
I(637)/I(321)
I(672)/I(321)
0.0
0.1
0.2
0.3
0.4
0.5
0.6
0.7
0.8
0.9
1.0
300
250
200
150
100

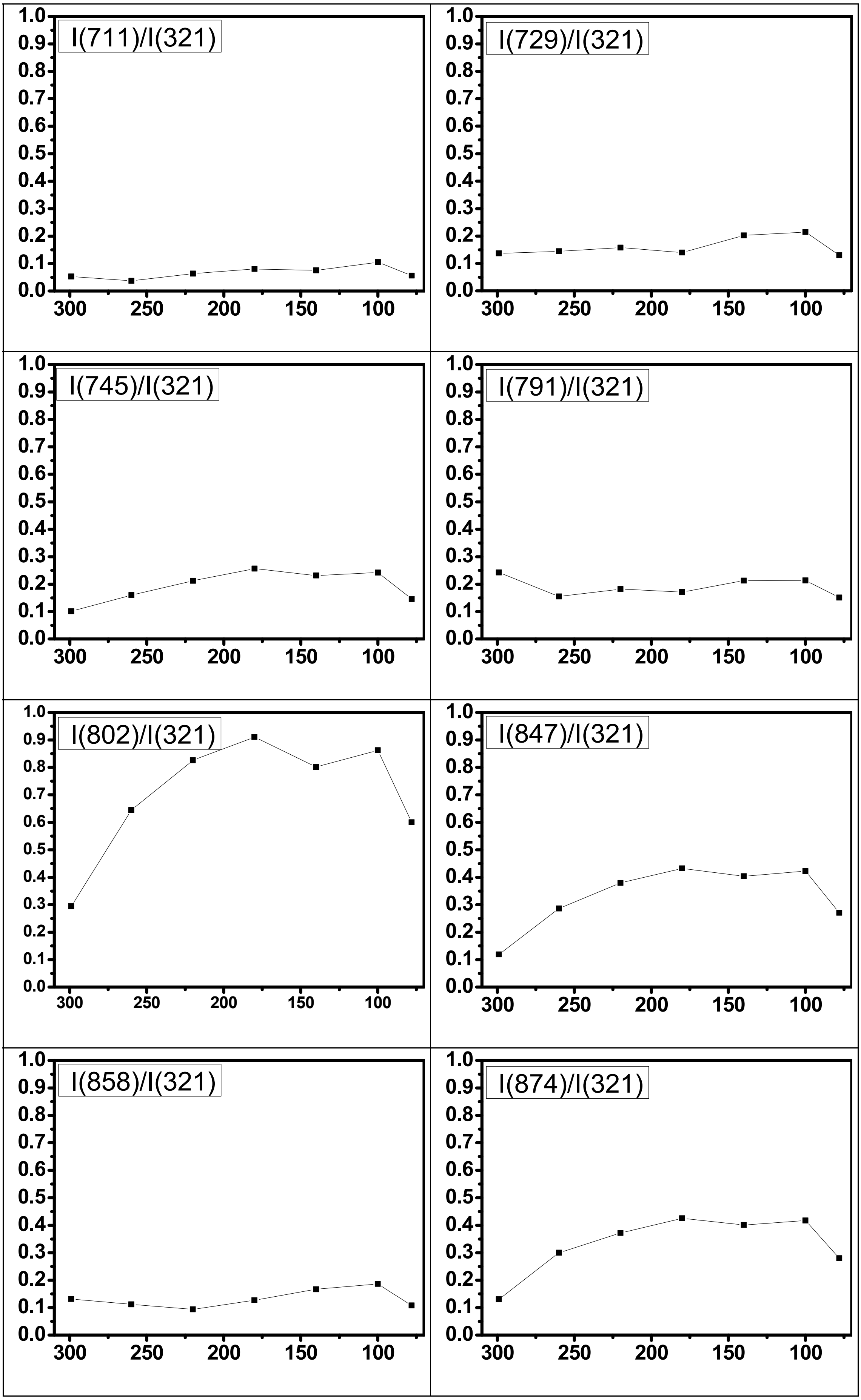
I(711)/I(321)
I(729)/I(321)
I(745)/I(321)
I(791)/I(321)
I(802)/I(321)
I(847)/I(321)
I(858)/I(321)
I(874)/I(321)
0.0 0.1 0.2 0.3 0.4 0.5 0.6 0.7 0.8 0.9 1.0
300 250 200 150 100

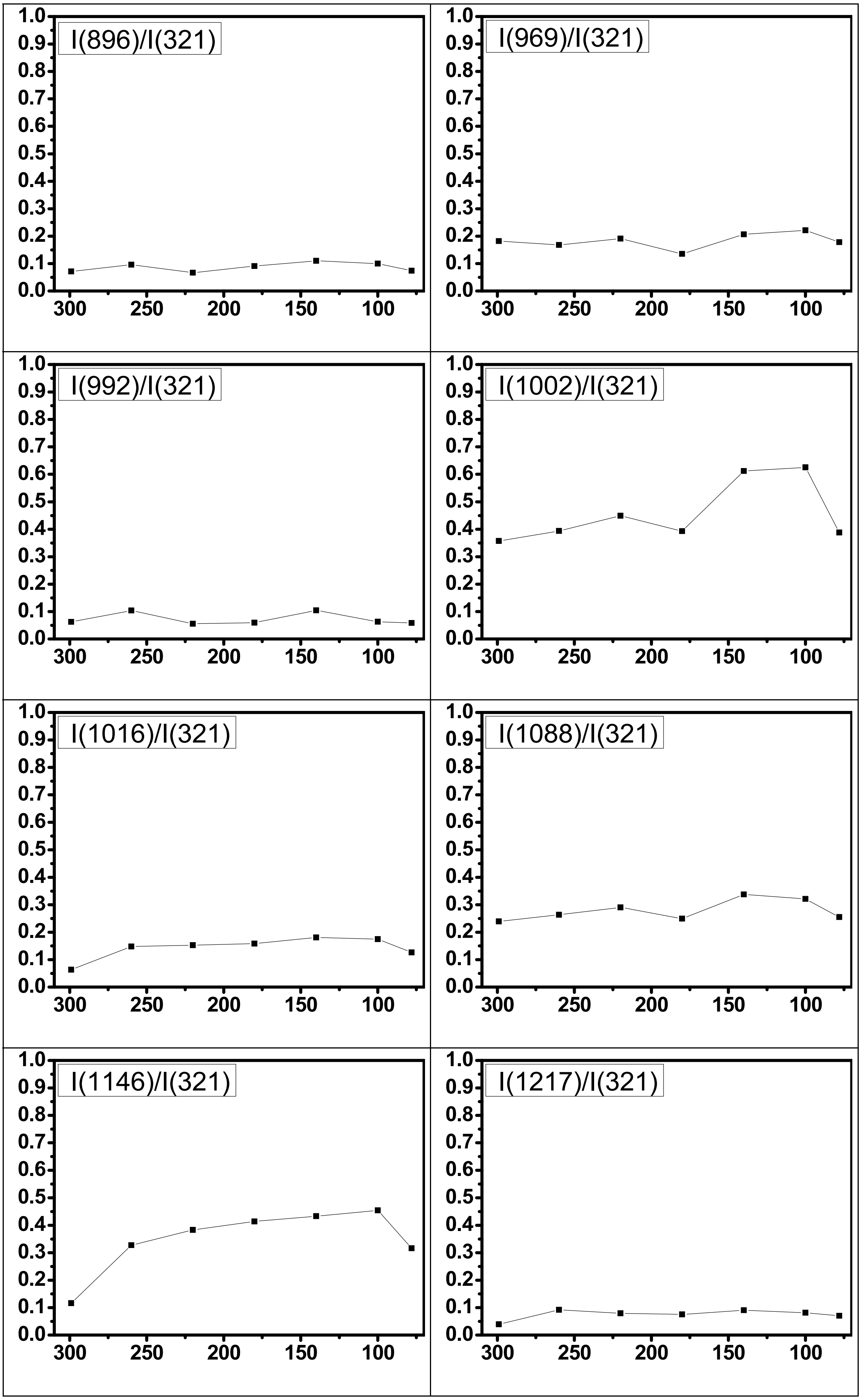
I(896)/I(321)
I(969)/I(321)
I(992)/I(321)
I(1002)/I(321)
I(1016)/I(321)
I(1088)/I(321)
I(1146)/I(321)
I(1217)/I(321)
0.0
0.1
0.2
0.3
0.4
0.5
0.6
0.7
0.8
0.9
1.0
300
250
200
150
100

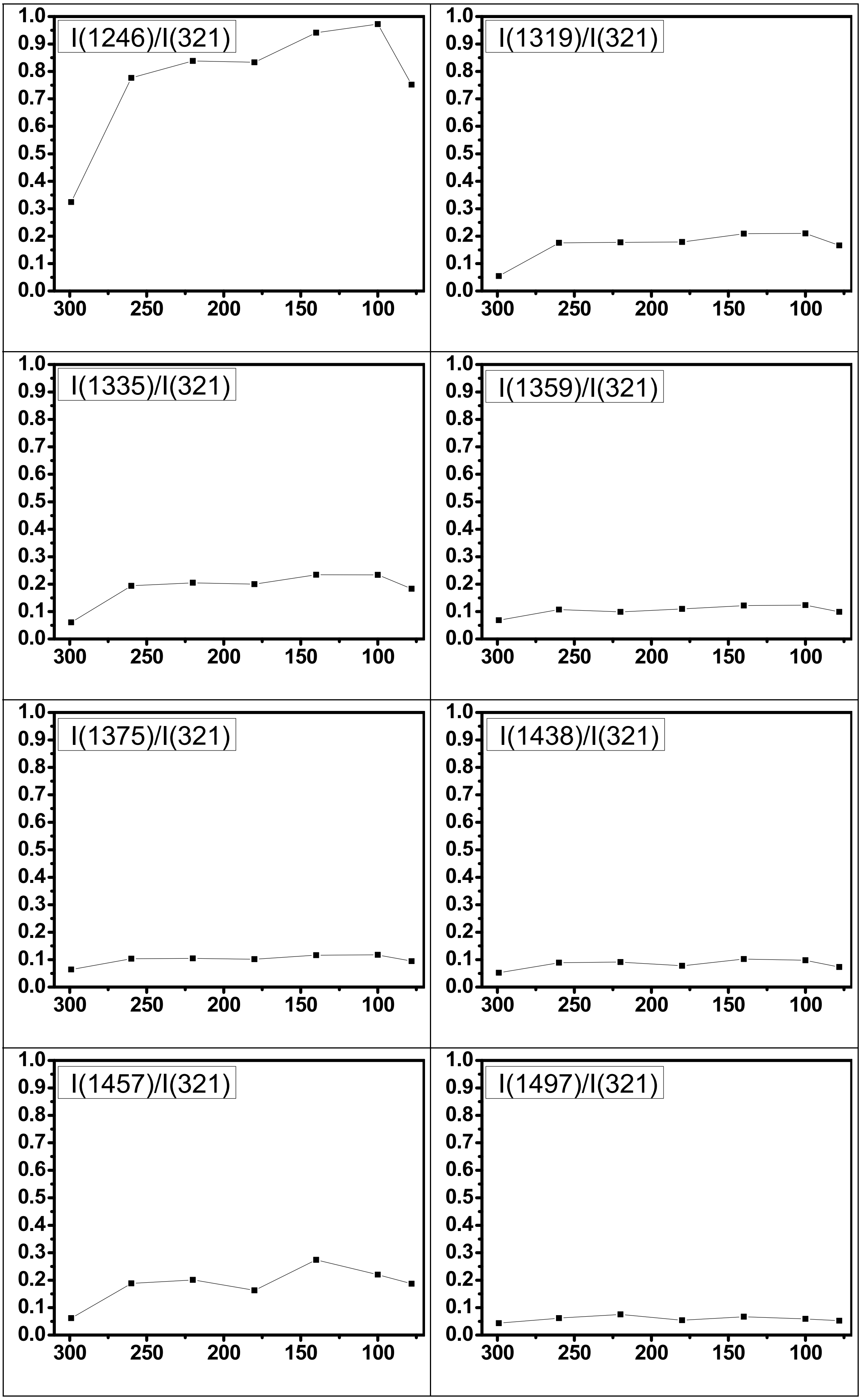
I(1246)/I(321)
I(1319)/I(321)
I(1335)/I(321)
I(1359)/I(321)
I(1375)/I(321)
I(1438)/I(321)
I(1457)/I(321)
I(1497)/I(321)
0.0 0.1 0.2 0.3 0.4 0.5 0.6 0.7 0.8 0.9 1.0
300 250 200 150 100

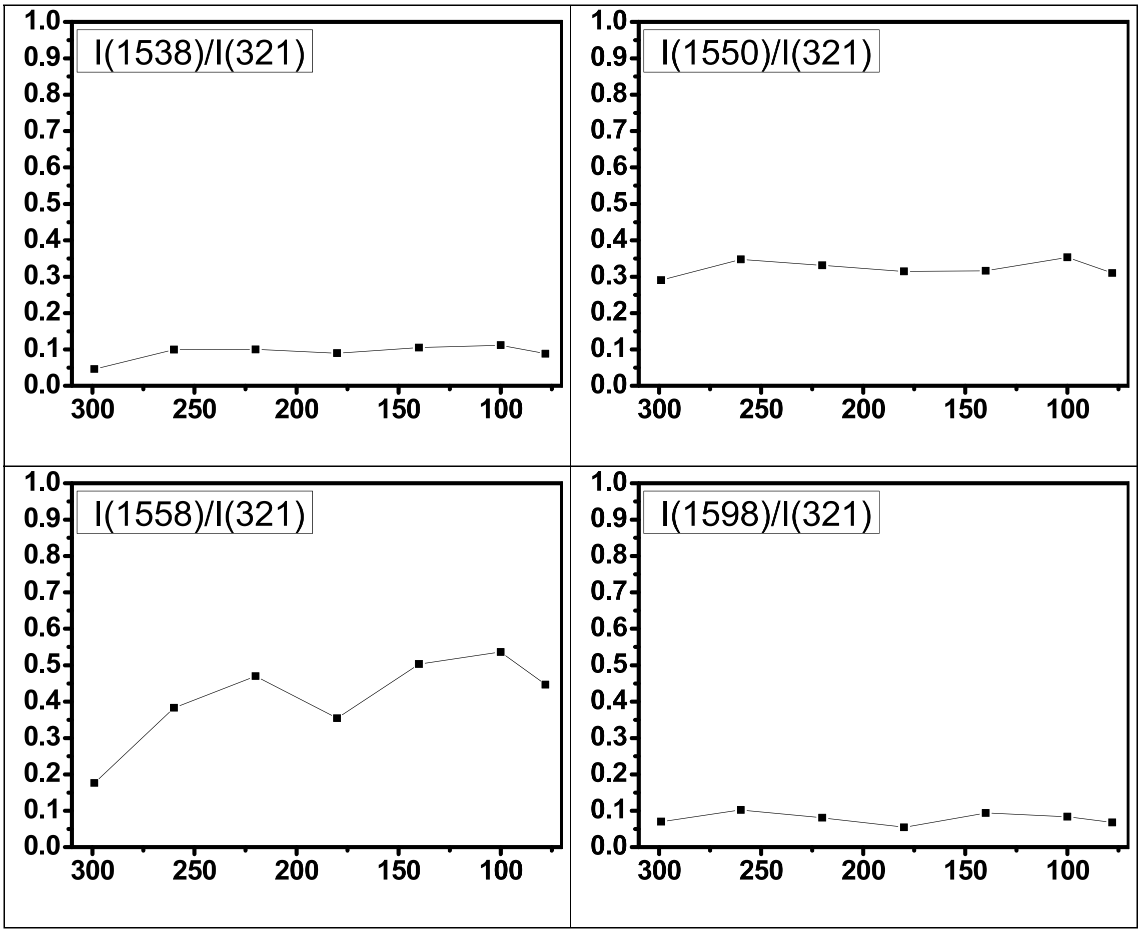
I(1538)/I(321)
I(1550)/I(321)
I(1558)/I(321)
I(1598)/I(321)
1.0
0.9
0.8
0.7
0.6
0.5
0.4
0.3
0.2
0.1
0.0
300
250
200
150
100
